# Network meta-analysis of treatment interventions for *Helicobacter pylori* infection in adult populations in East and Southeast Asia

**DOI:** 10.3389/fphar.2024.1462057

**Published:** 2024-10-10

**Authors:** Li Zhang, Bo-Ren Li, Si-Tong Guo, Yan Li

**Affiliations:** Department of Pharmacy, The People’s Hospital of Guangxi Zhuang Autonomous Region, Nanning, China

**Keywords:** *Helicobacter pylori*, treatment regimens, network meta-analysis (NMA), East and Southeast Asia, adult, eradication rate

## Abstract

**Background:**

*Helicobacter pylori* (*H. pylori*) infection poses a global health challenge, necessitating diverse treatment strategies. This network meta-analysis aimed to assess various treatment regimens for *H. pylori* in East and Southeast Asian populations.

**Methods:**

A systematic search was conducted in PubMed, Embase, and the Cochrane Library databases from inception to 20 Dec 2023, to identify relevant randomized controlled trials (RCTs) on *H. pylori* treatment interventions in East Asian and Southeast Asian populations. The primary outcome focused on effectiveness, specifically the rate of *H. pylori* eradication, while the secondary outcome evaluated overall safety, including the incidence of total and serious adverse effects. Network geometry plots were generated to illustrate direct and indirect treatment comparisons, using triple therapy (TT) as the reference standard. Odds Ratios (ORs) and 95% confidence intervals (CIs) were calculated using random-effects models to account for study heterogeneity and consistency models for indirect comparisons. The treatment hierarchy was assessed using the ranking probabilities and surface under the cumulative ranking curve (SUCRA) values.

**Results:**

79 studies met the inclusion criteria, with 99 paired comparisons. The included studies, conducted in Southeast Asia and among East Asian populations, included 29,903 patients. Significant outcomes in treat effectiveness were observed in various comparisons, such as sequential therapy vs. TT, bismuth quadruple therapy (BQT) vs. TT, high-dose dual therapy (HDDT) vs. TT, concomitant therapy vs. TT, P-CAB-based therapy vs. TT, and R-HT/HT vs. TT. R-HT/HT was the top choice based on rankograms and SUCRA values (SUCRA = 96.5). Regarding overall safety, significant results were noted in comparisons involving BQT, HDDT, concomitant therapy, sequential therapy, and P-CAB-based therapy. HDDT achieved the highest overall safety based on rankograms and SUCRA values (SUCRA = 0.0). HDDT demonstrated the lowest incidence of serious adverse events, according to global rankograms and SUCRA values (SUCRA = 19.7).

**Conclusion:**

This network meta-analysis highlights the complexity of treating *H. pylori* in East and Southeast Asia. R-HT/HT emerged as the most effective regimen, while HDDT proved to be the safest, with the lowest incidence of serious adverse events. These findings are crucial for optimizing treatment protocols in these regions.

**Systematic Review Registration:**

https://www.crd.york.ac.uk/prospero/display_record.php?ID=CRD42023435318.

## Introduction

*Helicobacter pylori* (*H. pylori*) infection remains a pressing global public health concern, affecting approximately 4.4 billion individuals worldwide (Martin-Nuñez et al., 2021). This infection is a primary cause of chronic gastritis, peptic ulcer, lymphoid tissue lymphoma associated with gastric mucosa, and, in particular, gastric cancer (Rokkas et al., 2021). The timely eradication of *H. pylori* facilitates the healing of peptic ulcers and significantly decreases the risk of ulcer complications, recurrence, and the development of gastric cancer (Ford et al., 2020). A comprehensive global systematic review conducted in 2015 revealed the highest prevalence of *H. pylori* in Africa (79.1%), Latin America and the Caribbean (63.4%), and Asia (54.7%). Within Asia, Southeast Asia represented 43.1% of cases (Hooi et al., 2017). In particular, the prevalent dietary habits in Asia contribute substantially to the increased incidence of *H. pylori* infections among its population.

However, no universally effective treatment can ideally address *H. pylori* in all populations (Talebi Bezmin Abadi, 2014). The Asia-Pacific Consensus Guidelines for *H. pylori* in 2009 currently recommend triple therapy based on proton-pump inhibitor (PPI) as first-line treatment for *H. pylori* eradication (Auesomwang et al., 2018), the 2023 Global Guideline on *H. pylori* also recommend this therapy (Kambara et al., 2020). This regimen incorporates amoxicillin and clarithromycin. Despite the widespread use of this approach, the increasing prevalence of antibiotic-resistant strains of *H. pylori* has resulted in lower success rates with traditional triple therapies, with some studies reporting eradication in less than 50% of cases (Jha et al., 2019). To address this challenge, many countries have developed tailored eradication regimens based on their specific conditions to improve the success rate of eradication of *H. pylori*. These regimens include bismuth quadruple, sequential, simultaneous, potassium-competitive acid blocker P-CAB-based, and R-hybrid therapies (O’Connor et al., 2020). Specifically, the P-CAB and R-HT/HT regimens were tested exclusively in Southeast Asian populations. However, the effectiveness and overall safety of these regimens warrant further investigation.

A network meta-analysis (NMA) evaluated the effectiveness of eight global first-line *H. pylori* eradication regimens. The results showed that vonoprazan (VPZ)-triple therapy and R-hybrid therapy achieved high eradication rates of over 90%. Levofloxacin triple therapy achieved the highest eradication rates in Western countries (Rokkas et al., 2021). This difference may be attributed to the various choices of eradication drugs in different regions, such as differences in resistance rates to clarithromycin, metronidazole, and levofloxacin. In addition, genetic factors leading to CYP2C19 gene polymorphism also contribute to efficacy. Traditionally, PPIs have been used to eradicate *H. pylori*. However, in recent years, new acid suppressants such as P-CAB have been used in many Asian countries and may be more effective than PPIs (Dong et al., 2017). In the present study, we conducted an NMA using up-to-date data from East Asia and Southeast Asia on first-line *H. pylori* eradication strategies and compared the rate of eradication and the incidence of adverse effects in various treatment strategies.

## Methods

### Search strategy and selection criteria

According to the previously established protocol (PROSPERO: CRD42023435318), the PRISMA 2020 checklist is presented in Supplementary Table S1. Ethical approval is not necessary for this study. We systematically searched the PubMed, Embase, and Cochrane Library databases from inception to 20 Dec 2023. Detailed search strategies are outlined in Supplementary Table S2. Furthermore, we identified potential studies in the reference lists of retrieved articles and unpublished data from ClinicalTrials.gov. Studies were included if they 1) were published as complete articles with extractable data; 2) involved Southeast Asian or East Asian populations; 3) were randomized controlled trials (RCTs) comparing different regimens; 4) had a minimum of 50 patients per arm; 5) administered therapeutic regimens as first-line treatments (the study subjects are first-time eradicators); 6) were written in English; and 7) included adult participants. Studies that did not meet these criteria, those with ‘double-counted’ patients, and studies in which drug susceptibility tests were performed in advance were excluded. Furthermore, RCTs that compared the same regimen in both arms but with different doses or durations of the included drugs were also excluded as they were irrelevant to the purposes of this study. Eligible studies were screened by two authors based on the above criteria.

### Study outcomes, data extraction, and quality assessment

The primary outcome focused on effectiveness, specifically the rate of *H. pylori* eradication, while the secondary outcome assessed overall safety, including the incidence of total and serious adverse effects. Serious adverse events are defined as those that cause significant harm to the patient or are intolerable and lead to discontinuation of the drug. Two authors (Li Zhang and Bo-Ren Li) independently extracted data using a predesigned format containing study characteristics, demographics, clinical characteristics and reported eradication rates. All information was extracted from the main text and the Supplementary Material. Only extractable data were analyzed. The methodological quality of the trials involved was evaluated using the Cochrane Collaboration Risk of Bias Tool (2.0) (Flemyng et al., 2023), We evaluated each study based on five domains, The studies were categorized into three levels. Disagreements about study selection, data extraction, and quality assessment processes were resolved through consultation with the corresponding investigator.

### Statistical analysis

A network geometry plot was generated to show direct- and indirect-treatment comparisons. This graph used triple therapy (TT) as a reference comparator for network assessments in different treatment regimens. Odds Ratios (ORs) and confidence intervals (95% CIs) were calculated using random-effects and consistency models. The ranking probability was used to establish a hierarchy of treatments. For primary and secondary outcomes, surface under the cumulative ranking curve (SUCRA) values were calculated to rank the treatments based on the cumulative probability plots. According to SUCRA, treatment regimens were ranked from worst (the lowest rate of *H. pylori* eradication) to best (the highest rate of eradication). Regarding overall safety, treatment regimens were ranked similarly from worst (the highest ADR rate) to best (the lowest ADR rate). Transitivity was evaluated to ensure consistency and coherence within the network. Interaction analyses were conducted to assess the comparability of results between consistency and inconsistency models, while node-splitting analyses were used to gauge coherence. All data were analyzed using STATA version 13.0 (Stata-Corp, College Station, Texas, United States).

## Results

### Characteristics and quality of the studies included

**FIGURE 1:**
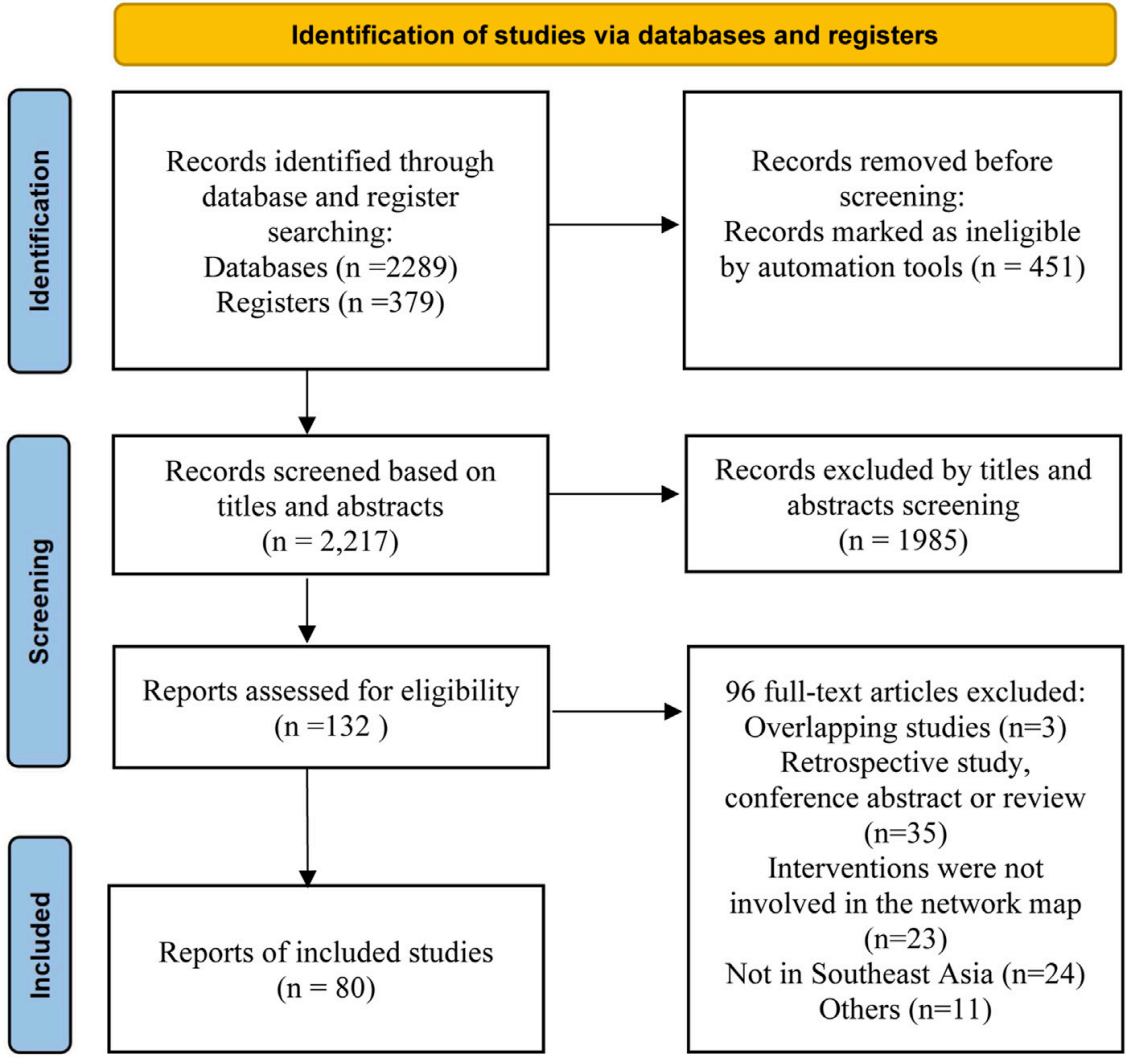
Flow chart of studies included in the network meta-analysis.

Our initial search yielded 2,217 articles. After screening the titles and abstracts, 1,985 articles were excluded. The remaining 132 records were subjected to a detailed review, as shown in Figure 1. Ultimately, 79 studies met the inclusion criteria. These comprised 68 two-arm and 11 three-arm RCTs, with 99 paired comparisons. The characteristics of these studies are presented in Supplementary Table S3. The included studies, conducted in Southeast Asia and among East Asian populations, included 29,903 patients. These patients were randomized to seven first-line treatment regimens: 1) bismuth quadruple therapy (BQT); 2) non-bismuth quadruple therapy (concomitant); 3) high-dose amoxicillin double treatment (HDDT); 4) P-CAB-based therapy; 5) reverse hybrid therapy (R-HT/HT); 6) sequential treatment; and 7) triple therapy (TT). The patient demographic and clinical characteristics are detailed in Supplementary Table S4. Regarding the quality of studies, several studies conducted double-blind trials. Although most other studies were single-blind or open-label, their randomization process was reasonable. As a result, the overall risk of bias for most studies is moderate or low. Among them, 3.8% are high-risk, while medium-risk and low-risk both account for 48.1%. Details on the quality assessment are given in Supplementary Table S5.

### Network Map

**FIGURE 2:**
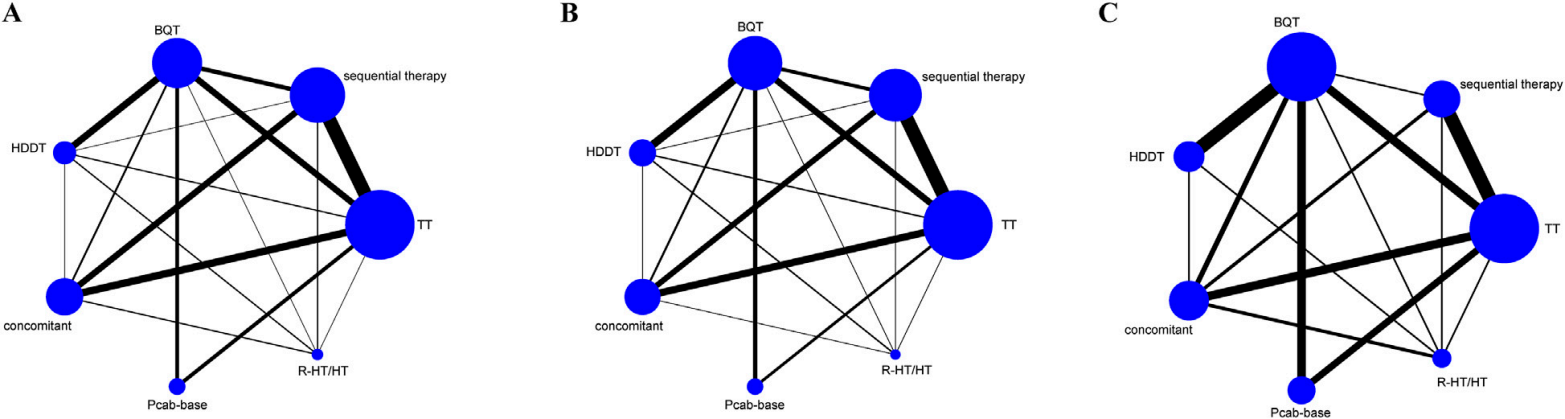
Network map in the randomized controlled trials (RCTs). **(A)** Effectiveness **(B)** Overall safety **(C)** Serious adverse events.

Figure 2A shows the network map of the seven therapeutic interventions (regimens) examined. This map shows the 21 potential comparisons, including 12 direct and 16 indirect comparisons between the regimens. Figure 2B shows the network map for the overall safety of these interventions. Figure 2C focuses on serious adverse drug reactions (ADRs). In this figure, the size of each node is directly proportional to the number of patients allocated to each treatment, and the thickness of the lines (edges) between nodes is proportional to the precision of the data, representing the inverse of the variance for each direct comparison.

### Clinical outcomes

#### Effectiveness

**FIGURE 3:**
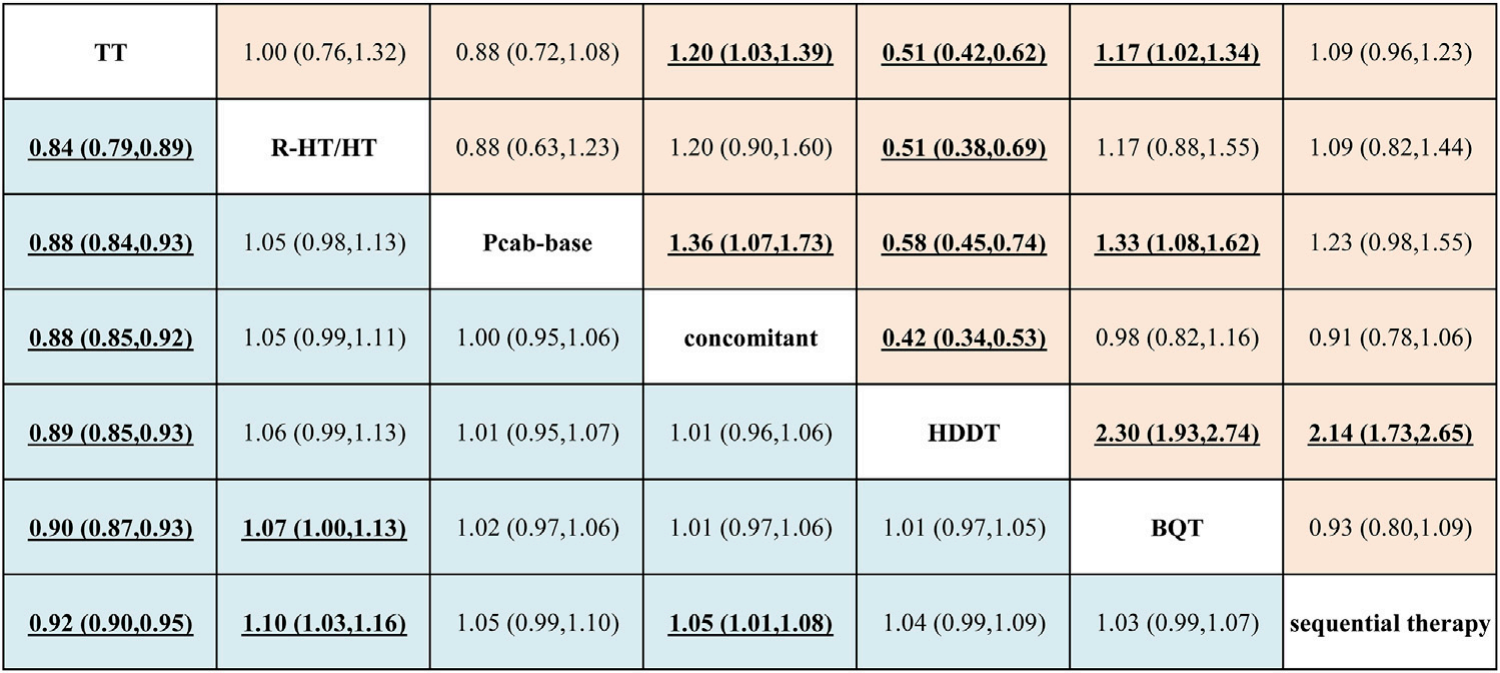
Effectiveness and safety league table.

The League table (Figure 3) and the network forest plot in Supplementary Figure S1A present odds ratios (ORs) with 95% credible intervals (CI) for the 21 direct and indirect comparisons. Among these, significant results were observed in the comparisons of sequential therapy *versus* TT (OR = 1.08; 95% CI = 1.05–1.12), BQT *versus* TT (OR = 1.11; 95% CI = 1.08–1.15), HDDT *versus* TT (OR = 1.12; 95% CI = 1.07–1.17), concomitant *versus* TT (OR = 1.13; 95% CI = 1.09–1.17), P-CAB-based therapy *versus* TT (OR = 1.13; 95% CI = 1.08–1.19), and R-HT/HT *versus* TT (OR = 1.19; 95% CI = 1.12–1.26). Additional significant results included concomitant *versus* sequential therapy (OR = 1.05; 95% CI = 1.01–1.08), R-HT/HT *versus* sequential therapy (OR = 1.10; 95% CI = 1.03–1.16), and R-HT/HT *versus* BQT (OR = 1.07; 95% CI = 1.00–1.13).

In contrast, the comparisons of BQT *versus* sequential therapy, HDDT *versus* sequential therapy, P-CAB-based therapy *versus* sequential therapy, HDDT *versus* BQT, concomitant *versus* BQT, P-CAB-based therapy *versus* BQT, concomitant *versus* HDDT, P-CAB-based therapy *versus* HDDT, R-HT/HT *versus* HDDT, P-CAB-based therapy *versus* concomitant, R-HT/HT *versus* concomitant, and R-HT/HT *versus* P-CAB-based therapy yielded insignificant results. Most inconsistency assessments revealed overall results that were not statistically significant, indicating consistency in the comparative effect sizes obtained by these comparisons. The relevant funnel plot in Supplementary Figure S2A exhibits symmetry, suggesting no evidence of publication bias or minor study effects.

#### Overall safety

The League table (Figure 3) and the network forest plot in Supplementary Figure S1B present ORs with 95% CIs for all 21 direct and indirect comparisons. Among these, significant results were observed in comparisons of BQT *versus* TT (OR = 1.17; 95% CI = 1.02–1.34), HDDT *versus* TT (OR = 0.51; 95% CI = 0.42–0.62), concomitant therapy *versus* TT (OR = 1.20; 95% CI = 1.03–1.39), HDDT *versus* sequential therapy (OR = 0.47; 95% CI = 0.38–0.58), HDDT *versus* BQT (OR = 0.43; 95% CI = 0.36–0.52), P-CAB-based therapy *versus* BQT (OR = 0.75; 95% CI = 0.62–0.92), concomitant *versus* HDDT (OR = 2.36; 95% CI = 1.88–2.96), P-CAB-based therapy *versus* HDDT (OR = 1.74; 95% CI = 1.34–2.24), R-HT/HT *versus* HDDT (OR = 1.97; 95% CI = 1.46–2.66), and P-CAB-based therapy *versus* concomitant therapy (OR = 0.74; 95% CI = 0.58–0.94).

In contrast, the comparisons of sequential therapy *versus* TT, P-CAB-based therapy *versus* TT, R-HT/HT *versus* TT, BQT *versus* sequential therapy, concomitant *versus* sequential therapy, R-HT/HT *versus* sequential therapy, concomitant *versus* BQT, R-HT/HT *versus* BQT, R-HT/HT *versus* concomitant, and R-HT/HT *versus* P-CAB-based therapy yielded insignificant results. The inconsistency evaluation showed overall results that were not statistically significant, indicating consistency in the comparative effect sizes obtained by these comparisons. The relevant funnel plot in Supplementary Figure S2B appears symmetrical, suggesting no evidence of publication bias or small study effects.

#### Serious adverse events

The network forest plot in Supplementary Figure S1C presents ORs with 95% CIs for the 21 direct and indirect comparisons. Among these, significant results were observed in the comparisons of BQT *versus* TT (OR = 2.27; 95% CI = 1.63–3.17), concomitant *versus* TT (OR = 1.82; 95% CI = 1.25–2.67), BQT *versus* sequential therapy (OR = 2.03; 95% CI = 1.24–3.31), HDDT *versus* BQT (OR = 0.38; 95% CI = 0.20–0.72), R-HT/HT *versus* BQT (OR = 0.47; 95% CI = 0.26–0.86), and concomitant *versus* HDDT (OR = 2.14; 95% CI = 1.04–4.39).

In contrast, the other comparisons yielded insignificant results. The inconsistency evaluation showed overall results that were not statistically significant, indicating consistency in the comparative effect sizes obtained by these comparisons. The relevant funnel plot in Supplementary Figure S2C appears symmetric, suggesting no evidence of publication bias or small study effects.

### Rankograms and SUCRA values

#### Effectiveness

**FIGURE 4:**
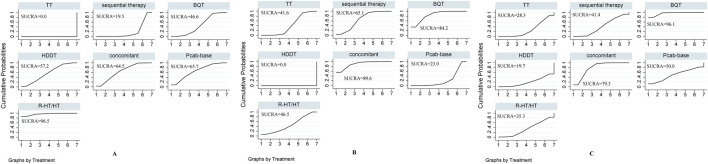
SUCRA-based rankograms for evaluated regimens in included RCTs. **(A)** Effectiveness **(B)** Overall safety **(C)** Serious adverse events.

Figure 4A shows all 21 comparisons and reveals that R-HT/HT exhibited the most favorable performance, followed in descending order of efficacy by P-CAB-based therapy, concomitant therapy, HDDT, BQT, sequential therapy, and TT, with TT being the least efficacious regimen. Consequently, based on the rankograms and SUCRA values, the overall results indicate that R-HT/HT, with a SUCRA value of 96.5%, is the top choice. This is followed by P-CAB-based therapy (SUCRA value 65.7%), concomitant therapy (SUCRA value 64.5%), HDDT (SUCRA value 57.2%), BQT (SUCRA value 46.6%), and sequential therapy (SUCRA value 19.5%). TT ranks as the least efficacious regimen (SUCRA value 0%).

#### Overall safety

Figure 4B shows all 21 comparisons, illustrating that HDDT exhibited the best safety performance, followed by P-CAB-based therapy, TT, R-HT/HT, sequential therapy, BQT, and concomitant therapy, the latter having the highest incidence of adverse effects among the regimens. Consequently, based on the rankograms and the SUCRA values, the global results indicate that HDDT (SUCRA value 0%) achieved the highest overall safety, followed by P-CAB-based therapy (SUCRA value 23%), TT (SUCRA value 41.6%), R-HT/HT (SUCRA value 46.5%), sequential therapy (SUCRA value 65.1%), and BQT (SUCRA value 84.2%). Concomitant therapy was ranked as the regimen with the lowest overall safety (SUCRA value 89.6%).

#### Serious adverse events

Figure 4C shows all 21 comparisons, revealing that HDDT performed best. It was followed by TT, R-HT/HT, sequential therapy, P-CAB-based therapy, concomitant therapy, and BQT, with BQT having the highest incidence of serious adverse events among the regimens. Therefore, based on rankograms and SUCRA values, the global results indicate that HDDT achieved the lowest incidence of serious ADRs (SUCRA value 19.7%). This was followed by TT (SUCRA value 28.3%), R-HT/HT (SUCRA value 35.3%), sequential therapy (SUCRA value 41.4%), P-CAB-base (SUCRA value 50%), and concomitant (SUCRA value 79.3%). BQT was ranked as the regimen with the highest incidence of serious ADRs (SUCRA value 96.1%).

## Discussion

The NMA efficacy results for *H. pylori* eradication indicated that the R-HT/HT treatment intervention exhibited relatively high effectiveness in East Asian and Southeast Asian populations. This was followed by P-CAB-base, concomitant, HDDT, BQT, and sequential therapy. TT showed the lowest eradication rate for *H. pylori*. This trend may be attributed to the increased resistance of *H. pylori* to antimicrobial agents, especially clarithromycin, levofloxacin, and metronidazole (Hu et al., 2017). TT has been applied for a prolonged period, leading to observable changes in *H. pylori* drug resistance over time (Zhou et al., 2022). Its extended use may contribute to decreased sensitivity, resulting in reduced efficacy. TT comprises only a combination of two antibacterial drugs, and this decrease in sensitivity could significantly affect treatment effectiveness.

Given the characteristics of *H. pylori*, only a limited number of antibacterial drugs are effective against it (Talebi Bezmin Abadi, 2014), and no single drug can effectively eliminate *H. pylori* (O’Morain et al., 2018). Typically, combination therapy is required. Therefore, under the separate use of PPI-based and P-CAB-based drugs, regimens incorporating a broader spectrum of antibacterial drugs tend to achieve higher rates of *H. pylori* eradication. Consequently, the R-HT/HT and concomitant regimens, which employ triple antibacterial drugs, exhibit relatively better efficacy. Although the HDDT regimen includes only amoxicillin as an antibacterial drug, its efficacy is not the lowest. This might be due to all treatment regimens considered to be first-line treatments (the study subjects are first-time eradicators), which reduces *H. pylori* exposure to previous treatment failures that could lead to drug resistance (Zhu et al., 2020). Additionally, HDDT effectiveness may be related to the relative rarity of the resistance of *H. pylori* to amoxicillin in certain regions, and high doses of amoxicillin administered can exceed the minimum inhibitory concentration (MIC) for *H. pylori*, contributing to its efficacy (Mei et al., 2022; Fernández-Salazar et al., 2022).

The P-CAB-base regimen ranked second in efficacy. Although the specific antibacterial drugs used in this regimen were not further classified, the results suggest that P-CAB may play a significant role in eliminating *H. pylori* (Kato et al., 2019; Simadibrata et al., 2022). The potential of P-CAB to counteract *H. pylori* antibacterial drug resistance warrants further investigation. However, when selecting treatment regimens, it is crucial to consider factors such as disease patterns, local antibiotic resistance rates, previous antibacterial drug exposure, and comprehensive access to drug treatments (Zhou et al., 2022).

Two network meta-analyses have compared different *H. pylori* treatment regimens specifically in mainland China and South Korea (Li et al., 2024; Jung et al., 2017). However, while these studies provide valuable insights, our study aims to encompass a broader scope across the entire East and Southeast Asian region. We believe that expanding the range of studies and treatment regimens considered can yield more comprehensive and reliable results.

To this end, our research includes additional protocols not covered in the previous NMAs, such as the R-HT/HT regimen, which has been extensively studied in Taiwan, and the P-CAB regimen, commonly used in Japan but not yet adopted in Korea. Notably, our findings show that both the R-HT/HT and P-CAB regimens demonstrate superior efficacy, underscoring the importance of including them in comparative analyses with other treatment options. By incorporating a wider array of studies and treatment protocols, our goal is to conduct a thorough and inclusive analysis of treatment options across the entire East and Southeast Asian region.

Regarding efficacy, our findings diverge from current guidelines. No existing guideline incorporates all seven treatment regimens analyzed, with some regimens, like R-HT/HT, being specific to Taiwan and China, and P-CAB regimens used primarily in Japan. Additionally, while China and Japan’s guidelines recommend BQT or P-CAB and bismuth, Korea’s guidelines list several options but exclude P-CAB. Notably, none of the guidelines from China, Japan, or Korea include the R-HT/HT regimen, which, according to our study, shows the highest efficacy for *H. pylori* eradication.

Regarding safety assessment, according to the data analyzed, the total ADR rate and the severe ADR rate of HDDT are the lowest, indicating its relatively good safety performance. This may be attributed to the fact that the HDDT regimen involves the fewest number of drugs, combining only two. In contrast, other regimens are triple or quadruple therapies. The total ADR rate for TT is third from the bottom, which aligns with the inference that fewer drug types used lead to a lower total ADR rate (Graham et al., 2018). The most common ADRs identified are diarrhea and taste disturbance (Al-Eidan et al., 2002).

However, this inference has limitations because the variability in the number of drug types used in the P-CAB-based regimen in different studies prevented a comprehensive analysis of the impact of the number of drug types on its total ADR rate. Furthermore, we observed that the safety performance of the P-CAB-based regimen, measured by the total ADR rate, is second only to HDDT and superior to other PPI-based regimens. Whether this indicates a higher safety profile for P-CAB than PPI requires further investigation (Simadibrata et al., 2022; Cheng et al., 2021), a notion supported by studies conducted in Japan (Kambara et al., 2020). In particular, the regimen with the highest total ADR rate is the concomitant regimen, while the regimen with the highest severe ADR rate is BQT. These two findings are not consistent and warrant further exploration.

It is important to note that in our study, severe ADRs include cases in which treatment was discontinued due to ADRs. In some cases, these discontinuations may not necessarily reflect the severity of the reactions but rather the patients’ unwillingness to continue treatment. This reluctance often stems from gastrointestinal discomfort, a common side effect of bismuth used in the BQT regimen. Therefore, while severe ADRs may not always signify significant harm to the body, they can lead to decreased patient compliance and, ultimately, treatment failure. Gastrointestinal ADRs, although relatively harmless, should be carefully considered. Despite initial concerns about bismuth safety, subsequent studies have indicated low systemic bioavailability and a rare occurrence of severe ADRs, making it relatively safe at regular doses (Tillman et al., 1996).

However, it is essential to acknowledge the presence of heterogeneity in the comparison of efficacy and safety, indicating variations in the results between different studies. This heterogeneity may arise from factors such as diverse research methodologies, patient population variations, and treatment regimens. Our findings diverge from certain previous studies. For example, some guidelines do not recommend a three-antibiotic combination regimen (Katelaris et al., 2023; Malfertheiner et al., 2022). These discrepancies could be related to variations in patient populations and local antimicrobial resistance patterns. Our study focused on East Asian and Southeast Asian populations, intentionally excluding other ethnic groups that exhibit significant differences. However, P-CAB-based, concomitant, and R-HT/HT regimens maintained robust efficacy in all examined studies.

To comprehensively assess the efficacy and safety of various treatment interventions, we recommend conducting additional clinical trials in the future. Specifically, more trials are needed to gather accurate data on treatment interventions that have not been extensively studied. Some data in our study were of relatively low quality; therefore, future studies should prioritize data quality control to enhance the precision of the analysis results. The variability of patient populations in different regions and countries can influence the efficacy of treatment interventions. Hence, in future studies, it is crucial to thoroughly consider patient-specific factors to assess the effectiveness and safety of various treatment interventions.

## Limitation

This study has several limitations. First, variations in methodological quality and study design across different studies can introduce bias into the pooled results. Additionally, significant differences in design aspects, such as sample size and measurement methods, among the included studies can contribute to heterogeneity, affecting both the interpretability and reliability of the findings. Furthermore, since this study is based on existing data, addressing specific potential confounders or interactions may not be feasible. Finally, the interpretation and inference of the overall situation may be limited by the quality and quantity of the included studies, which introduces certain limitations to the analysis results.

## Conclusion

In East Asian and Southeast Asian populations, the treatment intervention R-HT/HT demonstrated relatively high effectiveness, followed by P-CAB-base, concomitant, HDDT, BQT, and sequential therapy. Traditional TT exhibited the lowest *H. pylori* eradication rate. Regarding safety, HDDT showed the lowest total and severe ADR rates. Furthermore, the P-CAB-base regimen, as indicated by its total ADR rate, ranked second only to HDDT, outperforming other PPI-based regimens in safety.

## Data Availability

The original contributions presented in the study are included in the article/Supplementary Material, further inquiries can be directed to the corresponding author.
