# Supplementary material for: Network meta-analysis of treatment interventions for Helicobacter pylori infection in adult populations in East and Southeast Asia
Source: Front Pharmacol. 2024 Oct 10;15:1462057. doi: 10.3389/fphar.2024.1462057 (PMC11499982; doi:10.3389/fphar.2024.1462057)
Supplement: Supplementary file 1 [file DataSheet1.docx]

SUPPLEMENTAL FILE

# Title: Network Meta-analysis of Treatment Interventions for Helicobacter pylori Infection in East Asia and Southeast Asia Populations

**Contents**

[Table S1. Checklist of the PRISMA extension for network meta-analysis 2](#_Toc26177)

[Table S2. Search strategy 6](#_Toc21614)

[Table S3. Baseline characteristics of 79 studies 8](#_Toc27451)

[Table S4. Patient demographics and clinical characteristics of RCTs 15](#_Toc12490)

[Table S5. Quality assessment of RCTs 22](#_Toc30822)

[Table S6. SUCRA ranking of treatment regimens for effectiveness outcomes 25](#_Toc30248)

[Table S7. SUCRA ranking of treatment regimens for overall safety outcomes 26](#_Toc26319)

[Table S8. SUCRA ranking of treatment regimens for Serious adverse events outcomes 27](#_Toc24579)

[Table S9. Node–splitting analysis of network meta-analysis 28](#_Toc14369)

[Figure S1. Network forest plot based on Effectiveness (A), Overall safety (B), and Serious adverse events (C) 30](#_Toc13577)

[Figure S2.The relevant funnel plot based on Effectiveness (A), Overall safety (B), and Serious adverse events (C) 31](#_Toc32054)

[References 32](#_Toc15345)

# Table S1. Checklist of the PRISMA extension for network meta-analysis

| **Section and**  **Topic** | **Item #** | **Checklist item** | **Location**  **where item is reported** |
| --- | --- | --- | --- |
| **TITLE** | | |  |
| Title | 1 | Identify the report as a systematic review. | Title |
| **ABSTRACT** | | |  |
| Abstract | 2 | See the PRISMA 2020 for Abstracts checklist. | Abstract |
| **INTRODUCTION** | | |  |
| Rationale | 3 | Describe the rationale for the review in the context of existing knowledge. | Introduction |
| Objectives | 4 | Provide an explicit statement of the objective(s) or question(s) the review addresses. | Introduction |
| **METHODS** | | |  |
| Eligibility criteria | 5 | Specify the inclusion and exclusion criteria for the review and how studies were grouped for the syntheses. | Search strategy and selection criteria |
| Information  sources | 6 | Specify all databases, registers, websites, organisations, reference lists and other sources searched or consulted to identify studies. Specify the date when each source was last searched or consulted. | Search strategy and selection criteria |
| Search strategy | 7 | Present the full search strategies for all databases, registers and websites, including any filters and limits used. | Search strategy and selection criteria |
| Selection process | 8 | Specify the methods used to decide whether a study met the inclusion criteria of the review, including how many reviewers screened each record and each report retrieved, whether they worked independently, and if applicable, details of automation tools used in the process. | Study outcomes, data extraction, and quality assessment |
| Data collection  process | 9 | Specify the methods used to collect data from reports, including how many reviewers collected data from each report, whether they worked  independently, any processes for obtaining or confirming data from study investigators, and if applicable, details of automation tools used in the process. | Study outcomes, data extraction, and quality assessment |
| Data items | 10a | List and define all outcomes for which data were sought. Specify whether all results that were compatible with each outcome domain in each study were sought (e.g. for all measures, time points, analyses), and if not, the methods used to decide which results to collect. | Statistical analysis |
|  | 10b | List and define all other variables for which data were sought (e.g. participant and intervention characteristics, funding sources). Describe any assumptions made about any missing or unclear information. | Statistical analysis |
| Study risk of bias assessment | 11 | Specify the methods used to assess risk of bias in the included studies, including details of the tool(s) used, how many reviewers assessed each study and whether they worked independently, and if applicable, details of automation tools used in the process. | Statistical analysis |
| Effect measures | 12 | Specify for each outcome the effect measure(s) (e.g. risk ratio, mean difference) used in the synthesis or presentation of results. | Statistical analysis |
| Synthesis  methods | 13a | Describe the processes used to decide which studies were eligible for each synthesis (e.g. tabulating the study intervention characteristics and comparing against the planned groups for each synthesis (item #5)). | Statistical analysis |
|  | 13b | Describe any methods required to prepare the data for presentation or synthesis, such as handling of missing summary statistics, or data conversions. | Statistical analysis |
|  | 13c | Describe any methods used to tabulate or visually display results of individual studies and syntheses. | Statistical analysis |
|  | 13d | Describe any methods used to synthesize results and provide a rationale for the choice(s). If meta-analysis was performed, describe the model(s), method(s) to identify the presence and extent of statistical heterogeneity, and software package(s) used. | Statistical analysis |
|  | 13e | Describe any methods used to explore possible causes of heterogeneity among study results (e.g. subgroup analysis, meta-regression). | Statistical analysis |
|  | 13f | Describe any sensitivity analyses conducted to assess robustness of the synthesized results. | NA |
| Reporting bias  assessment | 14 | Describe any methods used to assess risk of bias due to missing results in a synthesis (arising from reporting biases). | Statistical analysis |
| Certainty  assessment | 15 | Describe any methods used to assess certainty (or confidence) in the body of evidence for an outcome. | Statistical analysis |

| **Section and**  **Topic** | **Item #** | **Checklist item** | **Location**  **where item is reported** |
| --- | --- | --- | --- |
| **RESULTS** | | |  |
| Study selection | 16a | Describe the results of the search and selection process, from the number of records identified in the search to the number of studies included in the review, ideally using a flow diagram. | Characteristics and quality of the studies included |
|  | 16b | Cite studies that might appear to meet the inclusion criteria, but which were excluded, and explain why they were excluded. | Characteristics and quality of the studies included |
| Study  characteristics | 17 | Cite each included study and present its characteristics. | Characteristics and quality of the studies included |
| Risk of bias in  studies | 18 | Present assessments of risk of bias for each included study. | Characteristics and quality of the studies included |
| Results of  individual studies | 19 | For all outcomes, present, for each study: (a) summary statistics for each group (where appropriate) and (b) an effect estimate and its precision (e.g. confidence/credible interval), ideally using structured tables or plots. | Network Map |
| Results of  syntheses | 20a | For each synthesis, briefly summarise the characteristics and risk of bias among contributing studies. | Clinical outcomes |
|  | 20b | Present results of all statistical syntheses conducted. If meta-analysis was done, present for each the summary estimate and its precision (e.g. confidence/credible interval) and measures of statistical heterogeneity. If comparing groups, describe the direction of the effect. | Clinical outcomes |
|  | 20c | Present results of all investigations of possible causes of heterogeneity among study results. | Clinical outcomes |
|  | 20d | Present results of all sensitivity analyses conducted to assess the robustness of the synthesized results. | Clinical outcomes |
| Reporting biases | 21 | Present assessments of risk of bias due to missing results (arising from reporting biases) for each synthesis assessed. | Clinical outcomes |
| Certainty of  evidence | 22 | Present assessments of certainty (or confidence) in the body of evidence for each outcome assessed. | Clinical outcomes |
| **DISCUSSION** | | |  |
| Discussion | 23a | Provide a general interpretation of the results in the context of other evidence. | Discussion |
|  | 23b | Discuss any limitations of the evidence included in the review. | Limitation |
|  | 23c | Discuss any limitations of the review processes used. | Discussion |
|  | 23d | Discuss implications of the results for practice, policy, and future research. | Discussion |
| **OTHER INFORMATION** | | |  |
| Registration and protocol | 24a | Provide registration information for the review, including register name and registration number, or state that the review was not registered. | Search strategy and selection criteria |
|  | 24b | Indicate where the review protocol can be accessed, or state that a protocol was not prepared. | NA |
|  | 24c | Describe and explain any amendments to information provided at registration or in the protocol. | Search strategy and selection criteria |
| Support | 25 | Describe sources of financial or non-financial support for the review, and the role of the funders or sponsors in the review. | Funding |
| Competing  interests | 26 | Declare any competing interests of review authors. | Conflict of interest |
| Availability of  data, code and  other materials | 27 | Report which of the following are publicly available and where they can be found: template data collection forms; data extracted from included studies; data used for all analyses; analytic code; any other materials used in the review. | NA |

Table S2. Search strategy used in Dec 20, 2023

| **Literature databases** | **Search items** | **Items found** |
| --- | --- | --- |
| PUBMED | (((helicobacter pylori [MeSH Terms] OR (helicobacter [All Fields] AND pylori [All Fields]) OR helicobacter pylori [All Fields] OR h pylori [All Fields])) AND ((((((therapy [Subheading] OR therapy [All Fields])) OR (treatment [All Fields])) OR (therapeutics [MeSH Terms])) OR (therapeutics [All Fields])) OR (therapy [Subheading]) OR (therapy [All Fields]) OR (therapeutics [All Fields]) OR (clinical protocols [MeSH Terms]) OR (clinical [All Fields] AND protocols [All Fields]) OR (clinical protocols [All Fields]) OR (regimen [All Fields]) AND (eradication [All Fields]))) AND ((((((((randomized controlled trial[Publication Type]) OR (controlled clinical trial[Publication Type])) OR (randomized[Title/Abstract])) OR (placebo[Title/Abstract])) OR ("Clinical Trials as Topic"[Mesh:NoExp])) OR (randomly[Title/Abstract])) OR (trial[Title/Abstract])) NOT (("Animals"[Mesh]) NOT ("Humans"[Mesh]))) | 2738 |
| EMBASE | (('helicobacter pylori'/exp OR helicobacter) AND pylori OR 'helicobacter pylori' OR 'h pylori') AND (eradica* OR treat* OR terap*) AND ('randomized controlled trial'/exp OR 'randomized controlled trial') | 3461 |
| COCHRANE | #1 helicobacter pylori  #2 (therapy OR treatment OR therapeutics OR clinical protocols OR regimen) AND eradication  #3 randomized controlled trial OR controlled clinical trial OR randomized OR placebo OR "Clinical Trials as Topic": noexp OR randomly OR trial NOT "Animals”: exp NOT "Humans": exp  #1 AND #2 AND #3 | 631 |
| Overall |  | **6830** |

Table S3. Baseline characteristics of 79 studies

| **NO.** | Study, year | NCT | Country | Arm | Treatment | Numbers | Durante (day) |
| --- | --- | --- | --- | --- | --- | --- | --- |
| 1 | Hsu et.al 2023^1^ | NCT03779074 | Taiwan | 1 | R-HT/HT | 306 | 14 |
|  |  |  |  | 2 | HDDT | 306 | 14 |
|  |  |  |  | 3 | BQT | 306 | 10 |
| 2 | Hu et.al 2023^2^ | NR | China | 1 | Pcab-base | 97 | 14 |
|  |  |  |  | 2 | BQT | 97 | 14 |
| 3 | Kim et.al 2023^3^ | NCT04674774 | Korea | 1 | Pcab-base | 105 | 14 |
|  |  |  |  | 2 | BQT | 106 | 14 |
| 4 | Liu et.al 2023^4^ | NR | China | 1 | HDDT | 422 | 14 |
|  |  |  |  | 2 | BQT | 424 | 14 |
| 5 | Lu et.al 2023^5^ | NCT04907747 | China | 1 | Pcab-base | 156 | 10 and 14 |
|  |  |  |  | 2 | BQT | 78 | 14 |
| 6 | Panigrahi et.al 2023^6^ | NR | India | 1 | sequential therapy | 99 | 10 |
|  |  |  |  | 2 | BQT | 98 | 14 |
|  |  |  |  | 3 | TT | 99 | 14 |
| 7 | Peng et.al 2023^7^ | NCT05196945 | China | 1 | Pcab-base | 158 | 14 |
|  |  |  |  | 2 | BQT | 158 | 14 |
| 8 | Qian et.al 2023^8^ | NR | China | 1 | Pcab-base | 250 | 10 |
|  |  |  |  | 2 | BQT | 125 | 10 |
| 9 | Tai et.al 2023^9^ | NCT05152004 | Taiwan | 1 | HDDT | 122 | 14 |
|  |  |  |  | 2 | R-HT/HT | 121 | 14 |
| 10 | Yang et.al 2023^10^ | NR | China | 1 | HDDT | 75 | 14 |
|  |  |  |  | 2 | BQT | 75 | 14 |
| 11 | Yun et.al 2023^11^ | NR | China | 1 | HDDT | 108 | 14 |
|  |  |  |  | 2 | BQT | 108 | 14 |
| 12 | Ang et.al 2022^12^ | NCT03908619 | Singapore | 1 | Pcab-base | 119 | 7 |
|  |  |  |  | 2 | TT | 125 | 14 |
| 13 | Choi et.al 2022^13^ | NCT03317223 | Korea | 1 | Pcab-base | 175 | 7 |
|  |  |  |  | 2 | TT | 175 | 7 |
| 14 | Guan et.al 2022^14^ | NR | China | 1 | HDDT | 350 | 14 |
|  |  |  |  | 2 | BQT | 350 | 14 |
| 15 | Hou et.al 2022^15^ | NCT03050359 | China/Korea/Taiwan/Philippines | 1 | Pcab-base | 263 | 14 |
|  |  |  |  | 2 | BQT | 268 | 14 |
| 16 | Mei et.al 2022^16^ | NR | China | 1 | HDDT | 170 | 14 |
|  |  |  |  | 2 | BQT | 170 | 14 |
| 17 | Shao et.al 2022^17^ | NR | China | 1 | HDDT | 120 | 14 |
|  |  |  |  | 2 | BQT | 120 | 14 |
| 18 | Shen et.al 2022^18^ | NR | China | 1 | HDDT | 496 | 14 |
|  |  |  |  | 2 | BQT | 475 | 14 |
| 19 | Raina et.al 2021^19^ | NR | India | 1 | concomitant | 151 | 10 |
|  |  |  |  | 2 | TT | 151 | 14 |
| 20 | Kim et.al 2021^20^ | NCT02557932 | Korea | 1 | BQT | 175 | 10 |
|  |  |  |  | 2 | TT | 177 | 7 |
| 21 | Bunchorntavakul et.al 2021^21^ | NR | Thailand | 1 | Pcab-base | 61 | 7 |
|  |  |  |  | 2 | TT | 61 | 14 |
| 22 | Hsu et.al 2020^22^ | NCT02646332 | Taiwan | 1 | R-HT/HT | 124 | 14 |
|  |  |  |  | 2 | concomitant | 124 | 14 |
| 23 | Hwong-Ruey Leow et.al 2020^23^ | NR | Malaysia | 1 | HDDT | 97 | 14 |
|  |  |  |  | 2 | TT | 94 | 14 |
| 24 | Song et.al 2020^24^ | NR | China | 1 | HDDT | 380 | 14 |
|  |  |  |  | 2 | BQT | 380 | 14 |
| 25 | Myint et.al 2020^25^ | NCT04132479 | Myanmar | 1 | concomitant | 156 | 14 |
|  |  |  |  | 2 | sequential therapy | 157 | 10 |
| 26 | Kim et.al 2019^26^ | NR | Korea | 1 | sequential therapy | 377 | 10 |
|  |  |  |  | 2 | concomitant | 383 | 10 |
|  |  |  |  | 3 | TT | 377 | 7 |
| 27 | Jha et.al 2019^27^ | NR | India | 1 | concomitant | 67 | 14 |
|  |  |  |  | 2 | TT | 71 | 14 |
| 28 | Chung et.al 2019^28^ | NR | Korea | 1 | concomitant | 68 | 14 |
|  |  |  |  | 2 | BQT | 68 | 14 |
| 29 | Tai et.al 2019^29^ | NR | Taiwan | 1 | HDDT | 120 | 14 |
|  |  |  |  | 2 | concomitant | 120 | 7 |
| 30 | Yang et.al 2019^30^ | NR | China | 1 | HDDT | 116 | 14 |
|  |  |  |  | 2 | BQT | 116 | 14 |
| 31 | Sue et.al 2018^31^ | NR | Japan | 1 | Pcab-base | 55 | 7 |
|  |  |  |  | 2 | TT | 51 | 7 |
| 32 | Auesomwang et.al 2018^32^ | NCT01888237 | Thailand | 1 | sequential therapy | 60 | 10 |
|  |  |  |  | 2 | TT | 60 | 10 |
| 33 | Leow et.al 2018^33^ | NR | Malaysia | 1 | BQT | 120 | 7 |
|  |  |  |  | 2 | TT | 244 | 7 and 14 |
| 34 | Choe et.al 2018^34^ | NR | Korea | 1 | concomitant | 135 | 10 |
|  |  |  |  | 2 | BQT | 135 | 14 |
| 35 | Liou et.al 2018^35^ | NCT03156855 | Taiwan | 1 | sequential therapy | 310 | 14 |
|  |  |  |  | 2 | BQT | 310 | 10 |
| 36 | Ashokkumar et.al 2017^36^ | NR | India | 1 | R-HT/HT | 60 | 14 |
|  |  |  |  | 2 | sequential therapy | 60 | 10 |
| 37 | Hu et.al 2017^37^ | NR | China | 1 | HDDT | 174 | 14 |
|  |  |  |  | 2 | BQT | 89 | 14 |
| 38 | Park et.al 2017^38^ | NCT02108184 | Korea | 1 | concomitant | 172 | 10 and 14 |
|  |  |  |  | 2 | sequential therapy | 169 | 10 and 14 |
| 39 | Su et.al 2017^39^ | NR | China | 1 | BQT | 180 | 7 |
|  |  |  |  | 2 | TT | 90 | 7 |
| 40 | Wu et.al 2017^40^ | NR | Taiwan | 1 | BQT | 81 | 7 |
|  |  |  |  | 2 | TT | 81 | 7 |
| 41 | Maruyama et.al 2017^41^ | NR | Japan | 1 | Pcab-base | 72 | 7 |
|  |  |  |  | 2 | TT | 69 | 7 |
| 42 | Murakami et.al 2016^42^ | NCT01505127 | Japan | 1 | Pcab-base | 329 | 7 |
|  |  |  |  | 2 | TT | 321 | 7 |
| 43 | Wu et.al 2016^43^ | NCT01906879 | China | 1 | concomitant | 540 | 10 |
|  |  |  |  | 2 | BQT | 540 | 10 |
|  |  |  |  | 3 | TT | 540 | 14 |
| 44 | Lee et.al 2016^44^ | NCT02159976 | China | 1 | sequential therapy | 195 | 10 |
|  |  |  |  | 2 | BQT | 195 | 14 |
| 45 | Kim et.al 2016^45^ | NR | Korea | 1 | sequential therapy | 306 | 10 |
|  |  |  |  | 2 | TT | 295 | 7 |
| 46 | Chung et.al 2016^46^ | NR | Korea | 1 | concomitant | 176 | 10 |
|  |  |  |  | 2 | sequential therapy | 170 | 10 |
|  |  |  |  | 3 | TT | 171 | 10 |
| 47 | Liou et.al 2016^47^ | NCT01607918 | Taiwan | 1 | sequential therapy | 650 | 10 |
|  |  |  |  | 2 | TT | 650 | 14 |
| 48 | Yang et.al 2016^48^ | NR | China | 1 | sequential therapy | 90 | 10 |
|  |  |  |  | 2 | BQT | 109 | 7 |
| 49 | Chen et.al 2015^49^ | NR | Taiwan | 1 | sequential therapy | 87 | 10 |
|  |  |  |  | 2 | R-HT/HT | 88 | 14 |
| 50 | Lee et.al 2015^50^ | NR | Korea | 1 | TT | 340 | 7 |
|  |  |  |  | 2 | concomitant | 170 | 7 |
|  |  |  |  | 3 | sequential therapy | 170 | 10 |
| 51 | Heo et.al 2015^51^ | NR | Korea | 1 | concomitant | 238 | 10 |
|  |  |  |  | 2 | R-HT/HT | 241 | 10 |
| 52 | Liao et.al 2015^52^ | NR | China | 1 | BQT | 101 | 10 |
|  |  |  |  | 2 | sequential therapy | 99 | 10 |
| 53 | Yang et.al 2015^53^ | NCT01163435 | Taiwan | 1 | HDDT | 150 | 14 |
|  |  |  |  | 2 | sequential therapy | 150 | 10 |
|  |  |  |  | 3 | TT | 150 | 7 |
| 54 | Tai et.al 2015^54^ | NR | China | 1 | concomitant | 100 | 7 |
|  |  |  |  | 2 | TT | 100 | 7 |
| 55 | Hsu et.al 2015^55^ | NCT02359435 | Taiwan | 1 | R-HT/HT | 220 | 12 |
|  |  |  |  | 2 | TT | 220 | 12 |
| 56 | Ang et.al 2015^56^ | NCT02092506 | Singapore | 1 | sequential therapy | 154 | 10 |
|  |  |  |  | 2 | concomitant | 153 | 10 |
|  |  |  |  | 3 | TT | 155 | 10 |
| 57 | Heo et.al 2014^57^ | NR | China | 1 | concomitant | 174 | 10 |
|  |  |  |  | 2 | TT | 174 | 10 |
| 58 | Hsu et.al 2014^58^ | NCT1769365 | Taiwan | 1 | concomitant | 102 | 7 |
|  |  |  |  | 2 | sequential therapy | 102 | 10 |
|  |  |  |  | 3 | TT | 103 | 7 |
| 59 | Zhou et.al 2014^59^ | NR | China | 1 | sequential therapy | 140 | 10 |
|  |  |  |  | 2 | TT | 140 | 10 |
| 60 | Xie et.al 2014^60^ | NR | China | 1 | TT | 360 | 7 and 10 |
|  |  |  |  | 2 | BQT | 360 | 7 and 10 |
| 61 | Lee et.al 2014^61^ | NCT01887249 | Korea | 1 | sequential therapy | 217 | 10 and 15 |
|  |  |  |  | 2 | TT | 115 | 7 |
| 62 | Liu et.al 2014^62^ | NR | Hong Kong | 1 | sequential therapy | 179 | 10 |
|  |  |  |  | 2 | BQT | 178 | 10 |
| 63 | Nasa et.al 2013^63^ | NR | India | 1 | sequential therapy | 111 | 10 |
|  |  |  |  | 2 | TT | 120 | 14 |
| 64 | Javid et.al 2013^64^ | NR | India | 1 | sequential therapy | 138 | 10 |
|  |  |  |  | 2 | TT | 134 | 10 |
| 65 | Liao et.al 2013^65^ | NCT01667718 | China | 1 | BQT | 80 | 14 |
|  |  |  |  | 2 | TT | 81 | 14 |
| 66 | Lim et.al 2013^66^ | NR | Korea | 1 | concomitant | 78 | 14 |
|  |  |  |  | 2 | sequential therapy | 86 | 14 |
| 67 | Liou et.al 2013^67^ | NCT01042184 | Taiwan | 1 | sequential therapy | 300 | 14 |
|  |  |  |  | 2 | TT | 600 | 10 and 14 |
| 68 | Choi et.al 2012^68^ | NR | Korea | 1 | sequential therapy | 115 | 10 |
|  |  |  |  | 2 | TT | 345 | 7 and 10 and 14 |
| 69 | Huang et.al 2012^69^ | NR | Taiwan | 1 | sequential therapy | 85 | 10 |
|  |  |  |  | 2 | concomitant | 84 | 10 |
| 70 | Yanai et.al 2012^70^ | NR | Japan | 1 | concomitant | 59 | 7 |
|  |  |  |  | 2 | TT | 60 | 7 |
| 71 | Qian et.al 2012^71^ | NR | China | 1 | sequential therapy | 231 | 10 |
|  |  |  |  | 2 | TT | 114 | 7 |
| 72 | Park et.al 2012^72^ | NR | Korea | 1 | sequential therapy | 162 | 10 |
|  |  |  |  | 2 | TT | 164 | 7 |
| 73 | Oh et.al 2012^73^ | NR | Korea | 1 | sequential therapy | 116 | 10 |
|  |  |  |  | 2 | TT | 130 | 7 |
| 74 | Chung et.al 2012^74^ | NCT01418300 | Korea | 1 | sequential therapy | 79 | 10 |
|  |  |  |  | 2 | TT | 80 | 10 |
| 75 | Kim et.al 2011^75^ | NR | Korea | 1 | sequential therapy | 205 | 10 |
|  |  |  |  | 2 | TT | 204 | 14 |
| 76 | Choi et.al 2011^76^ | NR | China | 1 | concomitant | 98 | 7 |
|  |  |  |  | 2 | TT | 197 | 7 |
| 77 | Zheng et.al 2010^77^ | NR | China | 1 | BQT | 85 | 10 |
|  |  |  |  | 2 | TT | 85 | 7 |
| 78 | Gao et.al 2010^78^ | NR | China | 1 | BQT | 72 | 10 |
|  |  |  |  | 2 | sequential therapy | 72 | 10 |
|  |  |  |  | 3 | TT | 71 | 7 |
| 79 | Wu et.al 2010^79^ | NR | Taiwan | 1 | concomitant | 115 | 10 |
|  |  |  |  | 2 | sequential therapy | 117 | 10 |

TT: triple therapy; BQT: bismuth quadruple therapy; R-HT/HT: R-hybrid therapy/hybrid therapy; HDDT: high-dose amoxicillin double treatment; Pcab: potassium-competitive acid blocker; NR: not report

Table S4. Patient demographics and clinical characteristics of RCTs

| **Study, year** | **Total number** | **Median**  **age** | **Female**  **(%)** | **Median BMI** | **Smoking (%)** | **Alcohol (%)** | **Peptic ulcer (%)** |
| --- | --- | --- | --- | --- | --- | --- | --- |
| Hsu et.al 2023 | 918 | 55 | 51 | NR | 21 | 12 | NR |
| Kim et.al 2023 | 211 | 58 | 43.1 | 25.1 | 32.7 | 50.2 | NR |
| Hu et.al 2023 | 194 | NR | 61.9 | NR | 13.9 | 43.8 | NR |
| Liu et.al 2023 | 846 | 41.2 | 49.3 | 23.7 | 15.4 | 17 | NR |
| Yang et.al 2023 | 150 | 66.9 | 60 | NR | 10 | 4.7 | NR |
| Peng et.al 2023 | 316 | 41 | 53.5 | 21.8 | 11.4 | 6.3 | NR |
| Qian et.al 2023 | 375 | 42.3 | 51.5 | 23.1 | 19.5 | 34.7 | NR |
| Lu et.al 2023 | 234 | 36.5 | 56 | 21.9 | NR | NR | NR |
| Tai et.al 2023 | 243 | 55.25 | 52.9 | NR | 10.1 | 11 | NR |
| Yun et.al 2023 | 216 | 48.2 | 28.1 | NR | NR | NR | NR |
| Panigrahi et.al 2023 | 296 | 42.35 | 28 | NR | NR | NR | 16.1 |
| Hou et.al 2022 | 531 | 41.7 | 35.8 | 22.85 | 31.9 | NR | 531 |
| Ang et.al 2022 | 244 | 52.3 | 38.1 | 24.95 | 14.8 | 17.6 | 6.6 |
| Guan et.al 2022 | 700 | 41.4 | 47.9 | 23.2 | 19.1 | 18.6 | NR |
| Mei et.al 2022 | 340 | 45 | 56.8 | 23.2 | 14.7 | 37.6 | NR |
| Choi et.al 2022 | 350 | 54 | 52 | 24.1 | 14.3 | 38 | 28.6 |
| Shen et.al 2022 | 971 | 46.4 | 57.9 | NR | NR | NR | NR |
| Shao et.al 2022 | 240 | 42.9 | 53.3 | 23.1 | 13.8 | 16.7 | NR |
| Raina et.al 2021 | 302 | 38.8 | 46.33 | NR | NR | NR | 34.8 |
| Kim et.al 2021 | 352 | 55.6 | 53.1 | NR | 34.5 | 59.4 | 13.9 |
| Bunchorntavakul et.al 2021 | 122 | 55.5 | 53.3 | 24.7 | 13.9 | 13.1 | 23 |
| Hsu et.al 2020 | 248 | 55.4 | 52.4 | NR | 17.7 | 4.8 | 47.6 |
| Hwong-Ruey Leow et.al 2020 | 191 | 56.7 | 54.5 | NR | 4.2 | NR | 4.7 |
| Song et.al 2020 | 760 | 41.3 | 53.7 | 23 | 17.1 | 18.7 | 11.2 |
| Myint et.al 2020 | 313 | 55 | 74 | NR | 25 | NR | NR |
| Tai et.al 2019 | 240 | 54.9 | 48.5 | NR | NR | NR | 11.4 |
| Yang et.al 2019 | 232 | 44 | 64.7 | 21.7 | 18.5 | 28.9 | NR |
| Kim et.al 2019 | 1137 | 55 | 45.3 | NR | 35.9 | 58.7 | NR |
| Jha et.al 2019 | 138 | 43.6 | 38.8 | NR | NR | NR | 43 |
| Chung et.al 2019 | 136 | 58.5 | 47.1 | 23.96 | 19.1 | 26.5 | 33.8 |
| Sue et.al 2018 | 106 | 63.2 | 32 | NR | 12.5 | NR | 6.1 |
| Auesomwang et.al 2018 | 120 | 54.9 | 61.7 | 24.5 | 17.5 | 30.8 | NR |
| Choe et.al 2018 | 270 | 54 | 36.3 | NR | 28.9 | NR | NR |
| Leow et.al 2018 | 364 | 55 | 51.9 | NR | 32.7 | NR | 4.4 |
| Liou et.al 2018 | 620 | 53.2 | 47.3 | 24.6 | 20.3 | 4.8 | NR |
| Wu et.al 2017 | 162 | 50.5 | 53.7 | NR | 26.5 | 9.9 | NR |
| Park et.al 2017 | 341 | 53.2 | 36.7 | 24.1 | 38.7 | 54.3 | 84.5 |
| Hu et.al 2017 | 263 | 46.1 | 60.5 | 22.5 | 19.7 | 18.3 | NR |
| Maruyama et.al 2017 | 141 | 58.9 | 42.6 | 22.5 | 25.5 | 31.2 | NR |
| Ashokkumar et.al 2017 | 120 | 40.4 | 53.3 | NR | NR | NR | NR |
| Su et.al 2017 | 270 | 43.1 | 50.4 | 22.8 | 24.1 | 23.3 | NR |
| Murakami et.al 2016 | 650 | 54.7 | 40 | 23 | NR | NR | NR |
| Wu et.al 2016 | 1620 | 53.4 | 50.6 | 24.4 | 10.6 | 8.5 | NR |
| Lee et.al 2016 | 390 | 53.4 | 43.6 | 23.7 | 18.9 | 37.7 | 34.9 |
| Kim et.al 2016 | 601 | 54 | 39.9 | NR | NR | NR | 88.5 |
| Chung et.al 2016 | 517 | 52.7 | 32.5 | NR | NR | NR | 86.1 |
| Liou et.al 2016 | 1300 | 49.3 | 50.5 | 24.5 | 19.1 | 31.2 | NR |
| Yang et.al 2016 | 199 | 48.7 | 51.3 | NR | NR | NR | 15.1 |
| Liao et.al 2015 | 200 | 41.9 | 46.4 | NR | 24 | 31 | NR |
| Yang et.al 2015 | 450 | 53.7 | 58.4 | 23.9 | 23 | 12.4 | 66.9 |
| Tai et.al 2015 | 200 | 50.3 | 50.5 | NR | 15.2 | 18.5 | 64.1 |
| Hsu et.al 2015 | 440 | 53 | 47 | NR | 19.5 | 4.5 | 47.3 |
| Ang et.al 2015 | 462 | 48.1 | 45 | NR | 22.5 | 26 | 12.1 |
| Chen et.al 2015 | 175 | 53.35 | 63.4 | NR | 11.4 | 21.7 | 44.6 |
| Heo et.al 2015 | 479 | 57.3 | 35.7 | NR | 29 | NR | NR |
| Lee et.al 2015 | 680 | 56.8 | 37.5 | NR | NR | NR | NR |
| Heo et.al 2014 | 348 | 57.9 | 37.9 | NR | 41.7 | NR | 27.3 |
| Hsu et.al 2014 | 307 | 55 | 43 | NR | 22.5 | 7.8 | 78.5 |
| Lee et.al 2014 | 332 | 54.8 | 49.4 | NR | NR | NR | 21.4 |
| Liu et.al 2014 | 357 | 56.4 | 56.6 | NR | 9.8 | 12.9 | NR |
| Zhou et.al 2014 | 280 | 43.5 | 52.9 | 22.6 | 13.9 | NR | NR |
| Xie et.al 2014 | 720 | 41 | 40 | NR | NR | NR | NR |
| Liao et.al 2013 | 161 | 47.8 | NR | NR | NR | NR | NR |
| Lim et.al 2013 | 164 | 56.8 | 46.3 | NR | 12.2 | 20.7 | NR |
| Liou et.al 2013 | 900 | 53.3 | NR | NR | 21.6 | 24.7 | 66.6 |
| Nasa et.al 2013 | 231 | 38.8 | 42.8 | NR | NR | NR | NR |
| Javid et.al 2013 | 272 | 38.2 | 38.6 | NR | 10.3 | NR | 100 |
| Yanai et.al 2012 | 119 | 61.5 | 21.8 | NR | NR | NR | NR |
| Qian et.al 2012 | 345 | 45.1 | 48.1 | 22.7 | 20.9 | 30.7 | NR |
| Park et.al 2012 | 326 | 52.8 | 46.6 | NR | 30.4 | NR | 43.9 |
| Oh et.al 2012 | 246 | 57.2 | 56.5 | NR | 8.1 | 19.1 | NR |
| Chung et.al 2012 | 159 | 49.6 | 43.1 | 23.6 | 32.1 | NR | 100 |
| Choi et.al 2012 | 460 | 46.8 | 48 | NR | NR | NR | NR |
| Huang et.al 2012 | 169 | 52.6 | 43.2 | NR | 32.5 | 22.5 | NR |
| Kim et.al 2011 | 409 | 51.2 | 48.2 | NR | 28.1 | 46 | 32.8 |
| Choi et.al 2011 | 295 | 54.9 | 42 | 24 | 23.4 | 36.6 | 80.7 |
| Zheng et.al 2010 | 170 | 42.1 | 55.3 | NR | NR | NR | NR |
| Gao et.al 2010 | 215 | 45 | 53.5 | NR | NR | NR | 80.5 |
| Wu et.al 2010 | 232 | 51.8 | 47.8 | NR | 22 | NR | NR |

CLA: clarithromycin; AMO: amoxicillin; MET: metronidazole; NR: not report.

Table S5. Quality assessment of RCTs

| **Study** | **Domain**  **signaling questions**  **bias arising from**  **the randomization**  **process** | **Bias due to**  **deviations**  **from intended**  **interventions** | **Bias due to missing**  **outcome data** | **Bias in**  **measurement of the outcome** | **Bias in selection of**  **the reported**  **result** | **Overall risk of bias judgement** |
| --- | --- | --- | --- | --- | --- | --- |
| Hsu et.al 2023 | Low | Low | Low | Low | Low | Low |
| Kim et.al 2023 | Low | Low | Low | Low | Low | Low |
| Hu et.al 2023 | Moderate | Low | Low | Low | Low | Moderate |
| Liu et.al 2023 | Low | Low | Low | Low | Low | Low |
| Yang et.al 2023 | Moderate | Low | Low | Low | Low | Moderate |
| Peng et.al 2023 | Low | Low | Low | Low | Low | Low |
| Qian et.al 2023 | Moderate | Low | Low | Low | Low | Moderate |
| Lu et.al 2023 | Moderate | Low | Low | Low | Low | Moderate |
| Tai et.al 2023 | Low | Low | Low | Low | Low | Low |
| Yun et.al 2023 | Moderate | Low | Low | Low | Low | Moderate |
| Panigrahi et.al 2023 | Low | Low | Low | Low | Low | Low |
| Hou et.al 2022 | Low | Low | Low | Low | Low | Low |
| Ang et.al 2022 | Low | Low | Low | Low | Low | Low |
| Guan et.al 2022 | Moderate | Low | Low | Low | Low | Moderate |
| Mei et.al 2022 | Moderate | Low | Low | Low | Low | Moderate |
| Choi et.al 2022 | Low | Low | Low | Low | Low | Low |
| Shen et.al 2022 | Moderate | Low | Low | Low | Low | Moderate |
| Shao et.al 2022 | Moderate | Low | Low | Low | Low | Moderate |
| Raina et.al 2021 | Moderate | Low | Low | Low | Low | Moderate |
| Kim et.al 2021 | Low | Low | Low | Low | Low | Low |
| Bunchorntavakul et.al 2021 | Moderate | Low | Low | Low | Low | Moderate |
| Hsu et.al 2020 | Low | Low | Low | Low | Low | Low |
| Hwong-Ruey Leow et.al 2020 | Moderate | Low | Low | Low | Low | Moderate |
| Song et.al 2020 | Low | Low | Low | Low | Low | Low |
| Myint et.al 2020 | Moderate | Low | Low | Low | Low | Moderate |
| Tai et.al 2019 | Moderate | Low | Low | Low | Low | Moderate |
| Yang et.al 2019 | Moderate | Low | Low | Low | Low | Moderate |
| Kim et.al 2019 | Low | Moderate | Moderate | Low | Low | High |
| Jha et.al 2019 | Moderate | Low | Low | Low | Low | Moderate |
| Chung et.al 2019 | Low | Low | Low | Low | Low | Low |
| Sue et.al 2018 | Moderate | Low | Low | Low | Low | Moderate |
| Auesomwang et.al 2018 | Low | Low | Low | Low | Low | Low |
| Choe et.al 2018 | High | Low | Low | Low | Low | High |
| Leow et.al 2018 | Moderate | Low | Low | Low | Low | Moderate |
| Liou et.al 2018 | Low | Low | Low | Low | Low | Low |
| Wu et.al 2017 | Moderate | Low | Low | Low | Low | Moderate |
| Park et.al 2017 | Low | Low | Low | Low | Low | Low |
| Hu et.al 2017 | Moderate | Low | Low | Low | Low | Moderate |
| Maruyama et.al 2017 | Moderate | Low | Low | Low | Low | Moderate |
| Ashokkumar et.al 2017 | Low | Low | Low | Low | Low | Low |
| Su et.al 2017 | Low | Low | Low | Low | Low | Low |
| Murakami et.al 2016 | Low | Low | Low | Low | Low | Low |
| Wu et.al 2016 | Low | Low | Low | Low | Low | Low |
| Lee et.al 2016 | Low | Low | Low | Low | Low | Low |
| Kim et.al 2016 | Moderate | Low | Low | Low | Low | Moderate |
| Chung et.al 2016 | Moderate | Low | Low | Low | Low | Moderate |
| Liou et.al 2016 | Low | Low | Low | Low | Low | Low |
| Yang et.al 2016 | Low | Low | Low | Low | Low | Low |
| Liao et.al 2015 | Moderate | Low | Low | Low | Low | Moderate |
| Yang et.al 2015 | Low | Low | Low | Low | Low | Low |
| Tai et.al 2015 | Low | Low | Low | Low | Low | Low |
| Hsu et.al 2015 | Low | Low | Low | Low | Low | Low |
| Ang et.al 2015 | Low | Low | Low | Low | Low | Low |
| Chen et.al 2015 | Moderate | Low | Low | Low | Low | Moderate |
| Heo et.al 2015 | Low | Low | Low | Low | Low | Low |
| Lee et.al 2015 | Moderate | Low | Low | Low | Low | Moderate |
| Heo et.al 2014 | Low | Low | Low | Low | Low | Low |
| Hsu et.al 2014 | Low | Low | Low | Low | Low | Low |
| Lee et.al 2014 | Moderate | Low | Low | Low | Low | Moderate |
| Liu et.al 2014 | Low | Low | Low | Low | Low | Low |
| Zhou et.al 2014 | Low | Low | Low | Low | Low | Low |
| Xie et.al 2014 | Moderate | Low | Low | Low | Low | Moderate |
| Liao et.al 2013 | Low | Low | Low | Low | Low | Low |
| Lim et.al 2013 | Moderate | Low | Low | Low | Low | Moderate |
| Liou et.al 2013 | Low | Low | Low | Low | Low | Low |
| Nasa et.al 2013 | Moderate | Low | Low | Low | Low | Moderate |
| Javid et.al 2013 | Low | Low | Low | Low | Low | Low |
| Yanai et.al 2012 | Moderate | Low | Low | Low | Low | Moderate |
| Qian et.al 2012 | Moderate | Low | Low | Low | Low | Moderate |
| Park et.al 2012 | Low | Low | Low | Low | Low | Low |
| Oh et.al 2012 | Moderate | Low | Low | Low | Low | Moderate |
| Chung et.al 2012 | Moderate | Low | Low | Low | Low | Moderate |
| Choi et.al 2012 | Moderate | Low | Low | Low | Low | Moderate |
| Huang et.al 2012 | Moderate | Low | Low | Low | Low | Moderate |
| Kim et.al 2011 | Low | Low | Low | Low | Low | Low |
| Choi et.al 2011 | Low | Low | Low | Low | Low | Low |
| Zheng et.al 2010 | High | Low | Low | Low | Low | High |
| Gao et.al 2010 | Moderate | Low | Low | Low | Low | Moderate |
| Wu et.al 2010 | Moderate | Low | Low | Low | Low | Moderate |

Low: low risk; Moderate: some concerns; High: high risk.

Table S6. SUCRA ranking of treatment regimens for effectiveness outcomes

| **Rank** | **TT** | **sequential therapy** | **BQT** | **HDDT** | **concomitant** | **Pcab-base** | **R-HT/HT** |
| --- | --- | --- | --- | --- | --- | --- | --- |
| best | 0.0 | 0.0 | 0.2 | 2.3 | 3.0 | 8.4 | 86.1 |
| 2nd | 0.0 | 0.0 | 4.1 | 20.3 | 32.6 | 33.6 | 9.4 |
| 3rd | 0.0 | 0.6 | 16.9 | 26.3 | 29.8 | 23.7 | 2.7 |
| 4th | 0.0 | 2.6 | 36.6 | 25.2 | 18.3 | 16.2 | 1.1 |
| 5th | 0.0 | 9.5 | 38.5 | 21.3 | 15.6 | 14.6 | 0.6 |
| 6th | 0.0 | 87.2 | 3.7 | 4.7 | 0.7 | 3.6 | 0.1 |
| Worst | 100.0 | 0.0 | 0.0 | 0.0 | 0.0 | 0.0 | 0.0 |

| **Treatment** | **SUCRA** | **PrBest** | **MeanRank** |
| --- | --- | --- | --- |
| TT | 0.0 | 0.0 | 7.0 |
| sequential therapy | 19.5 | 0.0 | 5.8 |
| BQT | 46.6 | 0.2 | 4.2 |
| HDDT | 57.2 | 2.3 | 3.6 |
| concomitant | 64.5 | 3.0 | 3.1 |
| Pcab-base | 65.7 | 8.4 | 3.1 |
| R-HT/HT | 96.5 | 86.1 | 1.2 |

SUCRA: the surface under the cumulative ranking curve; TT: triple therapy; BQT: bismuth quadruple therapy; R-HT/HT: R-hybrid therapy/hybrid therapy; HDDT: high-dose amoxicillin double treatment; Pcab: potassium-competitive acid blocker

Table S7. SUCRA ranking of treatment regimens for overall safety outcomes

| **Rank** | **TT** | **sequential therapy** | **BQT** | **HDDT** | **concomitant** | **Pcab-base** | **R-HT/HT** |
| --- | --- | --- | --- | --- | --- | --- | --- |
| best | 0.0 | 3.7 | 34.3 | 0.0 | 54.9 | 0.1 | 6.9 |
| 2nd | 0.4 | 17.7 | 42.0 | 0.0 | 31.3 | 0.4 | 8.2 |
| 3rd | 5.7 | 49.4 | 18.6 | 0.0 | 10.9 | 2.0 | 13.4 |
| 4th | 42.5 | 24.3 | 4.6 | 0.0 | 2.5 | 5.4 | 20.7 |
| 5th | 45.8 | 4.3 | 0.5 | 0.0 | 0.3 | 19.3 | 29.7 |
| 6th | 5.6 | 0.5 | 0.0 | 0.0 | 0.0 | 72.9 | 21.0 |
| Worst | 0.0 | 0.0 | 0.0 | 100.0 | 0.0 | 0.0 | 0.0 |

| **Treatment** | **SUCRA** | **PrBest** | **MeanRank** |
| --- | --- | --- | --- |
| TT | 41.6 | 0.0 | 4.5 |
| sequential therapy | 65.1 | 3.7 | 3.1 |
| BQT | 84.2 | 34.3 | 1.9 |
| HDDT | 0.0 | 0.0 | 7.0 |
| concomitant | 89.6 | 54.9 | 1.6 |
| Pcab-base | 23.0 | 0.1 | 5.6 |
| R-HT/HT | 46.5 | 6.9 | 4.2 |

SUCRA: the surface under the cumulative ranking curve; TT: triple therapy; BQT: bismuth quadruple therapy; R-HT/HT: R-hybrid therapy/hybrid therapy; HDDT: high-dose amoxicillin double treatment; Pcab: potassium-competitive acid blocker

Table S8. SUCRA ranking of treatment regimens for Serious adverse events outcomes

| **Rank** | **TT** | **sequential therapy** | **BQT** | **HDDT** | **concomitant** | **Pcab-base** | **R-HT/HT** |
| --- | --- | --- | --- | --- | --- | --- | --- |
| best | 0.0 | 0.1 | 78.4 | 0.1 | 8.2 | 12.7 | 0.5 |
| 2nd | 0.0 | 2.0 | 19.8 | 1.3 | 62.6 | 12.0 | 2.2 |
| 3rd | 3.6 | 20.7 | 1.8 | 7.2 | 25.9 | 23.3 | 17.5 |
| 4th | 18.0 | 30.5 | 0.1 | 12.0 | 3.0 | 13.3 | 23.2 |
| 5th | 34.9 | 23.6 | 0.0 | 12.8 | 0.2 | 9.7 | 18.7 |
| 6th | 31.5 | 15.8 | 0.0 | 20.0 | 0.0 | 11.7 | 21.1 |
| Worst | 12.0 | 7.3 | 0.0 | 46.5 | 0.0 | 17.3 | 16.8 |

| **Treatment** | **SUCRA** | **PrBest** | **MeanRank** |
| --- | --- | --- | --- |
| TT | 28.3 | 0.0 | 5.3 |
| sequential therapy | 41.4 | 0.1 | 4.5 |
| BQT | 96.1 | 78.4 | 1.2 |
| HDDT | 19.7 | 0.1 | 5.8 |
| concomitant | 79.3 | 8.2 | 2.2 |
| Pcab-base | 50.0 | 12.7 | 4.0 |
| R-HT/HT | 25.3 | 0.5 | 4.9 |

SUCRA: the surface under the cumulative ranking curve; TT: triple therapy; BQT: bismuth quadruple therapy; R-HT/HT: R-hybrid therapy/hybrid therapy; HDDT: high-dose amoxicillin double treatment; Pcab: potassium-competitive acid blocker

Table S9. Node–splitting analysis of network meta-analysis

| **Effectiveness** | **Direct effect** | | **Indirect effect** | |  |
| --- | --- | --- | --- | --- | --- |
| **Nodes** | **coefficient of β** | **SE** | **coefficient of β** | **SE** | **P** |
| TT, sequential therapy | 0.08 | 0.02 | 0.07 | 0.03 | 0.62 |
| TT, BQT | 0.08 | 0.03 | 0.13 | 0.02 | 0.12 |
| TT, HDDT | 0.13 | 0.05 | 0.11 | 0.03 | 0.82 |
| TT, concomitant | 0.14 | 0.03 | 0.09 | 0.03 | 0.17 |
| TT, Pcab-base | 0.12 | 0.03 | 0.13 | 0.04 | 0.84 |
| TT, R-HT/HT | 0.08 | 0.07 | 0.20 | 0.03 | 0.10 |
| sequential therapy, BQT | 0.02 | 0.03 | 0.04 | 0.02 | 0.72 |
| sequential therapy, HDDT | 0.11 | 0.07 | 0.03 | 0.03 | 0.25 |
| sequential therapy, concomitant | 0.04 | 0.03 | 0.05 | 0.03 | 0.97 |
| sequential therapy, R-HT/HT | 0.17 | 0.07 | 0.07 | 0.03 | 0.19 |
| BQT, HDDT | 0.01 | 0.02 | 0.01 | 0.04 | 0.97 |
| BQT, concomitant | -0.06 | 0.04 | 0.04 | 0.02 | 0.03 |
| BQT, Pcab-base | 0.02 | 0.03 | 0.01 | 0.04 | 0.84 |
| BQT, R-HT/HT | 0.01 | 0.06 | 0.08 | 0.03 | 0.39 |
| HDDT, concomitant | -0.05 | 0.07 | 0.01 | 0.03 | 0.44 |
| HDDT, R-HT/HT | 0.13 | 0.05 | 0.00 | 0.04 | 0.03 |
| concomitant, R-HT/HT | 0.01 | 0.05 | 0.07 | 0.04 | 0.34 |

TT: triple therapy; BQT: bismuth quadruple therapy; R-HT/HT: R-hybrid therapy/hybrid therapy; HDDT: high-dose amoxicillin double treatment; Pcab: potassium-competitive acid blocker

#

| **Overall safety** | **Direct effect** | | **Indirect effect** | |  |
| --- | --- | --- | --- | --- | --- |
| **Nodes** | **coefficient of β** | **SE** | **coefficient of β** | **SE** | **P** |
| TT, sequential therapy | 0.11 | 0.07 | 0.02 | 0.12 | 0.52 |
| TT, BQT | 0.05 | 0.11 | 0.25 | 0.10 | 0.16 |
| TT, HDDT | -0.64 | 0.22 | -0.69 | 0.12 | 0.85 |
| TT, concomitant | 0.15 | 0.10 | 0.24 | 0.12 | 0.54 |
| TT, Pcab-base | -0.04 | 0.14 | -0.23 | 0.15 | 0.37 |
| TT, R-HT/HT | 0.52 | 0.31 | -0.13 | 0.15 | 0.06 |
| sequential therapy, BQT | 0.25 | 0.15 | -0.00 | 0.09 | 0.14 |
| sequential therapy, HDDT | -0.73 | 0.29 | -0.77 | 0.12 | 0.90 |
| sequential therapy, concomitant | 0.07 | 0.10 | 0.14 | 0.12 | 0.65 |
| sequential therapy, R-HT/HT | -0.15 | 0.27 | -0.06 | 0.17 | 0.76 |
| BQT, HDDT | -0.83 | 0.10 | -0.86 | 0.18 | 0.87 |
| BQT, concomitant | 0.07 | 0.17 | 0.01 | 0.11 | 0.74 |
| BQT, Pcab-base | -0.36 | 0.13 | -0.17 | 0.16 | 0.37 |
| BQT, R-HT/HT | -0.17 | 0.27 | -0.15 | 0.17 | 0.95 |
| HDDT, concomitant | 0.86 | 0.41 | 0.86 | 0.12 | 1.00 |
| HDDT, R-HT/HT | 0.72 | 0.25 | 0.65 | 0.20 | 0.83 |
| concomitant, R-HT/HT | -0.65 | 0.31 | -0.05 | 0.16 | 0.09 |

TT: triple therapy; BQT: bismuth quadruple therapy; R-HT/HT: R-hybrid therapy/hybrid therapy; HDDT: high-dose amoxicillin double treatment; Pcab: potassium-competitive acid blocker

| **Serious adverse events** | **Direct effect** | | **Indirect effect** | |  |
| --- | --- | --- | --- | --- | --- |
| **Nodes** | **coefficient of β** | **SE** | **coefficient of β** | **SE** | **P** |
| TT, sequential therapy | 0.11 | 0.21 | 0.17 | 0.61 | 0.92 |
| TT, BQT | 0.93 | 0.18 | 0.17 | 0.46 | 0.13 |
| TT, concomitant | 0.53 | 0.24 | 0.76 | 0.37 | 0.61 |
| TT, Pcab-base | -0.33 | 0.89 | 0.39 | 0.66 | 0.70 |
| TT, R-HT/HT | 0.00 | 0.81 | 0.07 | 0.35 | 0.94 |
| sequential therapy, BQT | 0.01 | 0.80 | 0.78 | 0.26 | 0.36 |
| sequential therapy, concomitant | 0.72 | 0.80 | 0.46 | 0.28 | 0.75 |
| sequential therapy, R-HT/HT | -0.01 | 0.99 | -0.06 | 0.39 | 0.96 |
| BQT, HDDT | -0.94 | 0.34 | -1.71 | 1.13 | 0.49 |
| BQT, concomitant | -0.24 | 0.19 | -0.15 | 0.38 | 0.84 |
| BQT, Pcab-base | -0.44 | 0.63 | -0.86 | 0.91 | 0.70 |
| BQT, R-HT/HT | -0.36 | 0.58 | -0.91 | 0.36 | 0.42 |
| HDDT, concomitant | 0.00 | 1.99 | 0.79 | 0.37 | 0.70 |
| HDDT, R-HT/HT | 0.97 | 0.80 | -0.10 | 0.52 | 0.26 |
| concomitant, R-HT/HT | -0.79 | 0.40 | -0.23 | 0.45 | 0.36 |

TT: triple therapy; BQT: bismuth quadruple therapy; R-HT/HT: R-hybrid therapy/hybrid therapy; HDDT: high-dose amoxicillin double treatment; Pcab: potassium-competitive acid blocker.

Figure S1. Network forest plot based on Effectiveness (A), Overall safety (B), and Serious adverse events (C)


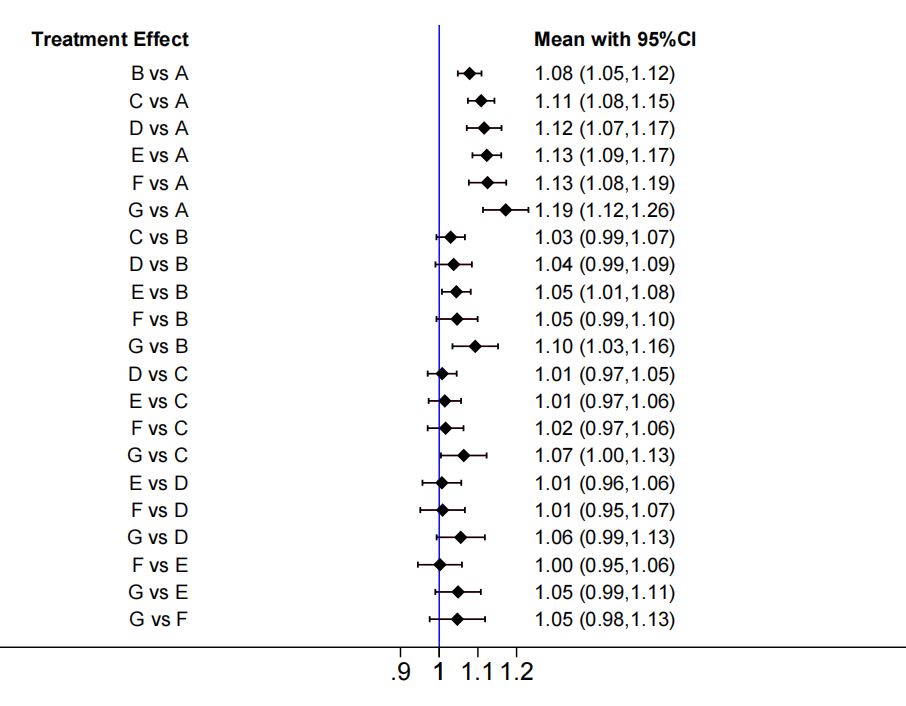

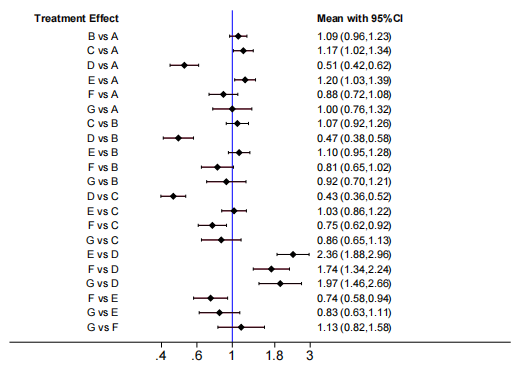

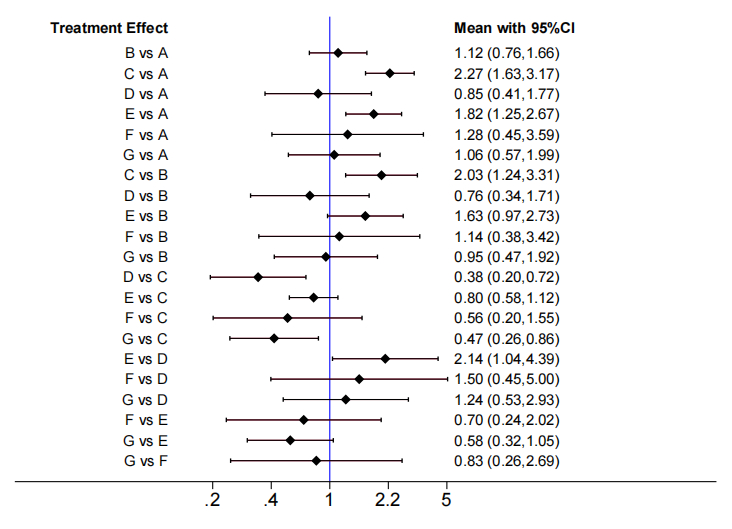


**A**

**B**

**C**

A: triple therapy; B: sequential therapy; C: bismuth quadruple therapy; D: high-dose amoxicillin double treatment; E: concomitant therapy; F: potassium-competitive acid blocker - base; G: R-hybrid therapy/hybrid therapy.

Figure S2. The relevant funnel plot based on Effectiveness (A), Overall safety (B), and Serious adverse events (C)


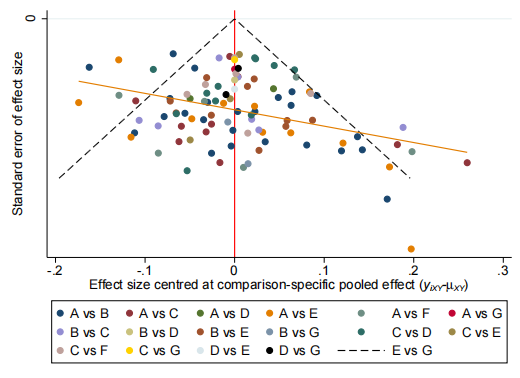

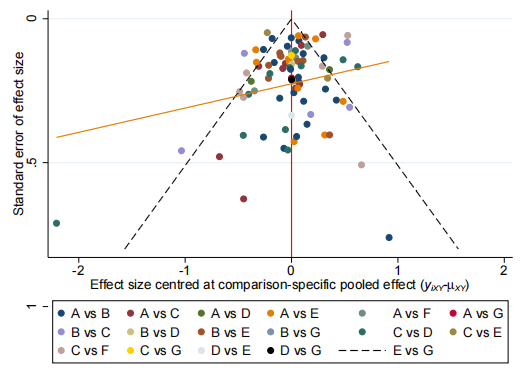

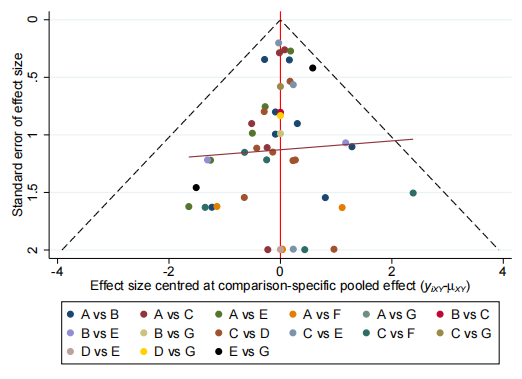


**A**

**B**

**C**

A: triple therapy; B: sequential therapy; C: bismuth quadruple therapy; D: high-dose amoxicillin double treatment; E: concomitant therapy; F: potassium-competitive acid blocker - base; G: R-hybrid therapy/hybrid therapy.

References

1. Hsu PI, Chen KY, Tai WC, et al. Hybrid, High-Dose Dual and Bismuth Quadruple Therapies for First-Line Treatment of Helicobacter pylori Infection in Taiwan: A Multicenter, Open-Label, Randomized Trial. *The American journal of gastroenterology* 2023.

2. Hu J, Mei H, Su NY, et al. Eradication rates of Helicobacter pylori in treatment-naive patients following 14-day vonoprazan-amoxicillin dual therapy: A multicenter randomized controlled trial in China. *Helicobacter* 2023: e12970.

3. Kim JS, Ko W, Chung JW, Kim TH. Efficacy of tegoprazan-based bismuth quadruple therapy compared with bismuth quadruple therapy for Helicobacter pylori infection: A randomized, double-blind, active-controlled study. *Helicobacter* 2023; **28**(3): e12977.

4. Liu DN, Wang QY, Li PY, et al. Comparing high-dose dual therapy with bismuth-containing quadruple therapy for the initial eradication of Helicobacter pylori infection on Hainan Island: A randomized, multicenter clinical trial. *Clinics and research in hepatology and gastroenterology* 2023; **47**(5): 102125.

5. Lu L, Wang Y, Ye J, et al. Quadruple therapy with vonoprazan 20 mg daily as a first-line treatment for Helicobacter pylori infection: A single-center, open-label, noninferiority, randomized controlled trial. *Helicobacter* 2023; **28**(1): e12940.

6. Panigrahi MK, Chouhan MI, Hallur VK, et al. Comparison of the efficacies of triple, quadruple and sequential antibiotic therapy in eradicating Helicobacter pylori infection: A randomized controlled trial. *Indian journal of gastroenterology : official journal of the Indian Society of Gastroenterology* 2023.

7. Peng X, Chen HW, Wan Y, et al. Combination of vonoprazan and amoxicillin as the first-line Helicobacter pylori eradication therapy: a multicenter, prospective, randomized, parallel-controlled study. *Clinical and experimental medicine* 2023.

8. Qian HS, Li WJ, Dang YN, et al. Ten-Day Vonoprazan-Amoxicillin Dual Therapy as a First-Line Treatment of Helicobacter pylori Infection Compared With Bismuth-Containing Quadruple Therapy. *The American journal of gastroenterology* 2023; **118**(4): 627-34.

9. Tai WC, Yang SC, Yao CC, et al. The Efficacy and Safety of 14-day Rabeprazole Plus Amoxicillin High Dose Dual Therapy by Comparing to 14-day Rabeprazole-Containing Hybrid Therapy for the Naïve Helicobacter pylori Infection in Taiwan: A Randomized Controlled Trial. *Infectious diseases and therapy* 2023.

10. Yang Q, He C, Hu Y, et al. 14-day pantoprazole- and amoxicillin-containing high-dose dual therapy for Helicobacter pylori eradication in elderly patients: A prospective, randomized controlled trial. *Frontiers in pharmacology* 2023; **14**: 1096103.

11. Yun JW, Wang C, Yu Y, et al. High-dose amoxicillin-proton pump inhibitor dual therapy as first-line treatment for Helicobacter pylori infection in Northwest China: A prospective, randomised controlled trial. *British journal of clinical pharmacology* 2023; **89**(1): 232-41.

12. Ang D, Koo SH, Chan YH, et al. Clinical trial: seven-day vonoprazan- versus 14-day proton pump inhibitor-based triple therapy for first-line Helicobacter pylori eradication. *Alimentary pharmacology & therapeutics* 2022; **56**(3): 436-49.

13. Choi YJ, Lee YC, Kim JM, et al. Triple Therapy-Based on Tegoprazan, a New Potassium-Competitive Acid Blocker, for First-Line Treatment of Helicobacter pylori Infection: A Randomized, Double-Blind, Phase III, Clinical Trial. *Gut and liver* 2022; **16**(4): 535-46.

14. Guan JL, Hu YL, An P, et al. Comparison of high-dose dual therapy with bismuth-containing quadruple therapy in Helicobacter pylori-infected treatment-naive patients: An open-label, multicenter, randomized controlled trial. *Pharmacotherapy* 2022; **42**(3): 224-32.

15. Hou X, Meng F, Wang J, et al. Vonoprazan non-inferior to lansoprazole in treating duodenal ulcer and eradicating Helicobacter pylori in Asian patients. *Journal of gastroenterology and hepatology* 2022; **37**(7): 1275-83.

16. Mei H, Guo Y, Zhao JT, et al. Efficacy and safety of high-dose esomeprazole and amoxicillin dual therapy versus bismuth-containing quadruple therapy for Helicobacter pylori infection: a multicenter, randomized controlled clinical trial. *Therapeutic advances in gastroenterology* 2022; **15**: 17562848221142925.

17. Shao QQ, Yu XC, Yu M, et al. Rabeprazole plus amoxicillin dual therapy is equally effective to bismuth-containing quadruple therapy for Helicobacter pylori eradication in central China: A single-center, prospective, open-label, randomized-controlled trial. *Helicobacter* 2022; **27**(2): e12876.

18. Shen C, Li C, Lv M, et al. The prospective multiple-centre randomized controlled clinical study of high-dose amoxicillin-proton pump inhibitor dual therapy for H. pylori infection in Sichuan areas. *Annals of medicine* 2022; **54**(1): 426-35.

19. Raina H, Sainani R, Parray A, Wani AH, Asharaf U, Raina MA. Efficacy of levofloxacin, omeprazole, nitazoxanide, and doxycycline (LOAD) regimen compared with standard triple therapy to eradicate Helicobacter pylori infection: a prospective randomized study from a tertiary hospital in India. *Gastroenterology and hepatology from bed to bench* 2021; **14**(4): 342-8.

20. Kim YI, Lee JY, Kim CG, Park B, Park JY, Choi IJ. Ten-day bismuth-containing quadruple therapy versus 7-day proton pump inhibitor-clarithromycin containing triple therapy as first-line empirical therapy for the Helicobacter pylori infection in Korea: a randomized open-label trial. *BMC gastroenterology* 2021; **21**(1): 95.

21. Bunchorntavakul C, Buranathawornsom A. Randomized clinical trial: 7-day vonoprazan-based versus 14-day omeprazole-based triple therapy for Helicobacter pylori. *Journal of gastroenterology and hepatology* 2021; **36**(12): 3308-13.

22. Hsu PI, Tsay FW, Kao JY, et al. Equivalent efficacies of reverse hybrid and concomitant therapies in first-line treatment of Helicobacter pylori infection. *Journal of gastroenterology and hepatology* 2020; **35**(10): 1731-7.

23. Hwong-Ruey Leow A, Chang JV, Goh KL. Searching for an optimal therapy for H pylori eradication: High-dose proton-pump inhibitor dual therapy with amoxicillin vs. standard triple therapy for 14 days. *Helicobacter* 2020; **25**(5): e12723.

24. Song Z, Zhou L, Xue Y, Suo B, Tian X, Niu Z. A comparative study of 14-day dual therapy (esomeprazole and amoxicillin four times daily) and triple plus bismuth therapy for first-line Helicobacter pylori infection eradication: A randomized trial. *Helicobacter* 2020; **25**(6): e12762.

25. Myint N, Zaw TT, Sain K, et al. Sequential Helicobacter pylori eradication therapy in Myanmar; a randomized clinical trial of efficacy and tolerability. *Journal of gastroenterology and hepatology* 2020; **35**(4): 617-23.

26. Kim BJ, Lee H, Lee YC, et al. Ten-Day Concomitant, 10-Day Sequential, and 7-Day Triple Therapy as First-Line Treatment for Helicobacter pylori Infection: A Nationwide Randomized Trial in Korea. *Gut and liver* 2019; **13**(5): 531-40.

27. Jha SK, Mishra MK, Saharawat K, Jha P, Purkayastha S, Ranjan R. Comparison of concomitant therapy versus standard triple-drug therapy for eradication of Helicobacter pylori infection: A prospective open-label randomized controlled trial. *Indian journal of gastroenterology : official journal of the Indian Society of Gastroenterology* 2019; **38**(4): 325-31.

28. Kim SJ, Chung JW, Woo HS, et al. Two-week bismuth-containing quadruple therapy and concomitant therapy are effective first-line treatments for Helicobacter pylori eradication: A prospective open-label randomized trial. *World journal of gastroenterology* 2019; **25**(46): 6790-8.

29. Tai WC, Liang CM, Kuo CM, et al. A 14 day esomeprazole- and amoxicillin-containing high-dose dual therapy regimen achieves a high eradication rate as first-line anti-Helicobacter pylori treatment in Taiwan: a prospective randomized trial. *The Journal of antimicrobial chemotherapy* 2019; **74**(6): 1718-24.

30. Yang X, Wang JX, Han SX, Gao CP. High dose dual therapy versus bismuth quadruple therapy for Helicobacter pylori eradication treatment: A systematic review and meta-analysis. *Medicine* 2019; **98**(7): e14396.

31. Sue S, Ogushi M, Arima I, et al. Vonoprazan- vs proton-pump inhibitor-based first-line 7-day triple therapy for clarithromycin-susceptible Helicobacter pylori: A multicenter, prospective, randomized trial. *Helicobacter* 2018; **23**(2): e12456.

32. Auesomwang C, Maneerattanaporn M, Chey WD, Kiratisin P, Leelakusolwong S, Tanwandee T. Ten-day high-dose proton pump inhibitor triple therapy versus sequential therapy for Helicobacter pylori eradication. *Journal of gastroenterology and hepatology* 2018; **33**(11): 1822-8.

33. Leow AH, Azmi AN, Loke MF, Vadivelu J, Graham DY, Goh KL. Optimizing first line 7-day standard triple therapy for Helicobacter pylori eradication: Prolonging treatment or adding bismuth: which is better? *Journal of digestive diseases* 2018; **19**(11): 674-7.

34. Choe JW, Jung SW, Kim SY, et al. Comparative study of Helicobacter pylori eradication rates of concomitant therapy vs modified quadruple therapy comprising proton-pump inhibitor, bismuth, amoxicillin, and metronidazole in Korea. *Helicobacter* 2018; **23**(2): e12466.

35. Liou JM, Chen PY, Luo JC, et al. Efficacies of Genotypic Resistance-Guided vs Empirical Therapy for Refractory Helicobacter pylori Infection. *Gastroenterology* 2018; **155**(4): 1109-19.

36. Ashokkumar S, Agrawal S, Mandal J, Sureshkumar S, Sreenath GS, Kate V. Hybrid Therapy versus Sequential Therapy for Eradication of Helicobacter pylori: A Randomized Controlled Trial. *Journal of pharmacology & pharmacotherapeutics* 2017; **8**(2): 62-7.

37. Hu JL, Yang J, Zhou YB, Li P, Han R, Fang DC. Optimized high-dose amoxicillin-proton-pump inhibitor dual therapies fail to achieve high cure rates in China. *Saudi journal of gastroenterology : official journal of the Saudi Gastroenterology Association* 2017; **23**(5): 275-80.

38. Park SM, Kim JS, Kim BW, Ji JS, Choi H. Randomized clinical trial comparing 10- or 14-day sequential therapy and 10- or 14-day concomitant therapy for the first line empirical treatment of Helicobacter pylori infection. *Journal of gastroenterology and hepatology* 2017; **32**(3): 589-94.

39. Su J, Zhou X, Chen H, Hao B, Zhang W, Zhang G. Efficacy of 1st-line bismuth-containing quadruple therapies with levofloxacin or clarithromycin for the eradication of Helicobacter pylori infection: A 1-week, open-label, randomized trial. *Medicine* 2017; **96**(7): e5859.

40. Wu TS, Hsu PI, Kuo CH, et al. Comparison of 10-day levofloxacin bismuth-based quadruple therapy and levofloxacin-based triple therapy for Helicobacter pylori. *Journal of digestive diseases* 2017; **18**(9): 537-42.

41. Maruyama M, Tanaka N, Kubota D, et al. Vonoprazan-Based Regimen Is More Useful than PPI-Based One as a First-Line Helicobacter pylori Eradication: A Randomized Controlled Trial. *Canadian journal of gastroenterology & hepatology* 2017; **2017**: 4385161.

42. Murakami K, Sakurai Y, Shiino M, Funao N, Nishimura A, Asaka M. Vonoprazan, a novel potassium-competitive acid blocker, as a component of first-line and second-line triple therapy for Helicobacter pylori eradication: a phase III, randomised, double-blind study. *Gut* 2016; **65**(9): 1439-46.

43. Wu DC, Kuo CH, Tsay FW, Hsu WH, Chen A, Hsu PI. A Pilot Randomized Controlled Study of Dexlansoprazole MR-Based Triple Therapy for Helicobacter Pylori Infection. *Medicine* 2016; **95**(11): e2698.

44. Lee YC, Chiang TH, Chou CK, et al. Association Between Helicobacter pylori Eradication and Gastric Cancer Incidence: A Systematic Review and Meta-analysis. *Gastroenterology* 2016; **150**(5): 1113-24.e5.

45. Kim SH, Yun JM, Chang CB, Piao H, Yu SJ, Shin DW. Prevalence of upper gastrointestinal bleeding risk factors among the general population and osteoarthritis patients. *World journal of gastroenterology* 2016; **22**(48): 10643-52.

46. Chung JW, Han JP, Kim KO, et al. Ten-day empirical sequential or concomitant therapy is more effective than triple therapy for Helicobacter pylori eradication: A multicenter, prospective study. *Digestive and liver disease : official journal of the Italian Society of Gastroenterology and the Italian Association for the Study of the Liver* 2016; **48**(8): 888-92.

47. Liou JM, Wu MS, Lin JT. Treatment of Helicobacter pylori infection: Where are we now? *Journal of gastroenterology and hepatology* 2016; **31**(12): 1918-26.

48. Yang X, Tan P, Song L, Lu Z. Comparison Between Sequential Therapy and Modified Bismuth-Included Quadruple Therapy for Helicobacter pylori Eradication in Chinese Patients. *American journal of therapeutics* 2016; **23**(6): e1436-e41.

49. Chen KY, Lin TJ, Lin CL, Lee HC, Wang CK, Wu DC. Hybrid vs sequential therapy for eradication of Helicobacter pylori in Taiwan: A prospective randomized trial. *World journal of gastroenterology* 2015; **21**(36): 10435-42.

50. Lee SW, Kim HJ, Kim JG. Treatment of Helicobacter pylori Infection in Korea: A Systematic Review and Meta-analysis. *Journal of Korean medical science* 2015; **30**(8): 1001-9.

51. Heo J, Jeon SW, Jung JT, et al. Concomitant and hybrid therapy for Helicobacter pylori infection: A randomized clinical trial. *Journal of gastroenterology and hepatology* 2015; **30**(9): 1361-6.

52. Liao XM, Nong GH, Chen MZ, et al. Modified sequential therapy vs quadruple therapy as initial therapy in patients with Helicobacter infection. *World journal of gastroenterology* 2015; **21**(20): 6310-6.

53. Yang JC, Lin CJ, Wang HL, et al. High-dose dual therapy is superior to standard first-line or rescue therapy for Helicobacter pylori infection. *Clinical gastroenterology and hepatology : the official clinical practice journal of the American Gastroenterological Association* 2015; **13**(5): 895-905.e5.

54. Tai WC, Liang CM, Lee CH, et al. Seven-Day Nonbismuth Containing Quadruple Therapy Could Achieve a Grade "A" Success Rate for First-Line Helicobacter pylori Eradication. *BioMed research international* 2015; **2015**: 623732.

55. Hsu PI, Lin PC, Graham DY. Hybrid therapy for Helicobacter pylori infection: A systemic review and meta-analysis. *World journal of gastroenterology* 2015; **21**(45): 12954-62.

56. Ang TL, Fock KM, Song M, et al. Ten-day triple therapy versus sequential therapy versus concomitant therapy as first-line treatment for Helicobacter pylori infection. *Journal of gastroenterology and hepatology* 2015; **30**(7): 1134-9.

57. Heo J, Jeon SW, Jung JT, et al. A randomised clinical trial of 10-day concomitant therapy and standard triple therapy for Helicobacter pylori eradication. *Digestive and liver disease : official journal of the Italian Society of Gastroenterology and the Italian Association for the Study of the Liver* 2014; **46**(11): 980-4.

58. Hsu PI, Wu DC, Chen WC, et al. Randomized controlled trial comparing 7-day triple, 10-day sequential, and 7-day concomitant therapies for Helicobacter pylori infection. *Antimicrobial agents and chemotherapy* 2014; **58**(10): 5936-42.

59. Zhou L, Lin S, Ding S, et al. Relationship of Helicobacter pylori eradication with gastric cancer and gastric mucosal histological changes: a 10-year follow-up study. *Chinese medical journal* 2014; **127**(8): 1454-8.

60. Xie Y, Zhu Y, Zhou H, et al. Furazolidone-based triple and quadruple eradication therapy for Helicobacter pylori infection. *World journal of gastroenterology* 2014; **20**(32): 11415-21.

61. Lee JW, Kim N, Kim JM, et al. A comparison between 15-day sequential, 10-day sequential and proton pump inhibitor-based triple therapy for Helicobacter pylori infection in Korea. *Scandinavian journal of gastroenterology* 2014; **49**(8): 917-24.

62. Liu KS, Hung IF, Seto WK, et al. Ten day sequential versus 10 day modified bismuth quadruple therapy as empirical firstline and secondline treatment for Helicobacter pylori in Chinese patients: an open label, randomised, crossover trial. *Gut* 2014; **63**(9): 1410-5.

63. Nasa M, Choksey A, Phadke A, Sawant P. Sequential therapy versus standard triple-drug therapy for Helicobacter pylori eradication: a randomized study. *Indian journal of gastroenterology : official journal of the Indian Society of Gastroenterology* 2013; **32**(6): 392-6.

64. Javid G, Zargar SA, Bhat K, et al. Efficacy and safety of sequential therapy versus standard triple therapy in Helicobacter pylori eradication in Kashmir India: a randomized comparative trial. *Indian journal of gastroenterology : official journal of the Indian Society of Gastroenterology* 2013; **32**(3): 190-4.

65. Liao J, Zheng Q, Liang X, et al. Effect of fluoroquinolone resistance on 14-day levofloxacin triple and triple plus bismuth quadruple therapy. *Helicobacter* 2013; **18**(5): 373-7.

66. Lim JH, Lee DH, Choi C, et al. Clinical outcomes of two-week sequential and concomitant therapies for Helicobacter pylori eradication: a randomized pilot study. *Helicobacter* 2013; **18**(3): 180-6.

67. Liou JM, Chen CC, Chen MJ, et al. Sequential versus triple therapy for the first-line treatment of Helicobacter pylori: a multicentre, open-label, randomised trial. *Lancet (London, England)* 2013; **381**(9862): 205-13.

68. Choi HS, Chun HJ, Park SH, et al. Comparison of sequential and 7-, 10-, 14-d triple therapy for Helicobacter pylori infection. *World journal of gastroenterology* 2012; **18**(19): 2377-82.

69. Huang J, Gong ST, Ou WJ, et al. [A 10-day sequential therapy for eradication of Helicobacter pylori infection in children]. *Zhonghua er ke za zhi = Chinese journal of pediatrics* 2012; **50**(8): 563-7.

70. Yanai A, Sakamoto K, Akanuma M, Ogura K, Maeda S. Non-bismuth quadruple therapy for first-line Helicobacter pylori eradication: A randomized study in Japan. *World journal of gastrointestinal pharmacology and therapeutics* 2012; **3**(1): 1-6.

71. Qian J, Ye F, Zhang J, et al. Levofloxacin-containing triple and sequential therapy or standard sequential therapy as the first line treatment for Helicobacter pylori eradication in China. *Helicobacter* 2012; **17**(6): 478-85.

72. Park HG, Jung MK, Jung JT, et al. Randomised clinical trial: a comparative study of 10-day sequential therapy with 7-day standard triple therapy for Helicobacter pylori infection in naïve patients. *Alimentary pharmacology & therapeutics* 2012; **35**(1): 56-65.

73. Oh HS, Lee DH, Seo JY, et al. Ten-day sequential therapy is more effective than proton pump inhibitor-based therapy in Korea: a prospective, randomized study. *Journal of gastroenterology and hepatology* 2012; **27**(3): 504-9.

74. Chung JW, Jung YK, Kim YJ, et al. Ten-day sequential versus triple therapy for Helicobacter pylori eradication: a prospective, open-label, randomized trial. *Journal of gastroenterology and hepatology* 2012; **27**(11): 1675-80.

75. Kim SY, Jung SW. [Helicobacter pylori eradication therapy in Korea]. *The Korean journal of gastroenterology = Taehan Sohwagi Hakhoe chi* 2011; **58**(2): 67-73.

76. Choi KH, Chung WC, Lee KM, et al. Efficacy of levofloxacin and rifaximin based quadruple therapy in Helicobacter pylori associated gastroduodenal disease: a double-blind, randomized controlled trial. *Journal of Korean medical science* 2011; **26**(6): 785-90.

77. Zheng Q, Chen WJ, Lu H, Sun QJ, Xiao SD. Comparison of the efficacy of triple versus quadruple therapy on the eradication of Helicobacter pylori and antibiotic resistance. *Journal of digestive diseases* 2010; **11**(5): 313-8.

78. Gao XZ, Qiao XL, Song WC, Wang XF, Liu F. Standard triple, bismuth pectin quadruple and sequential therapies for Helicobacter pylori eradication. *World journal of gastroenterology* 2010; **16**(34): 4357-62.

79. Wu DC, Hsu PI, Wu JY, et al. Sequential and concomitant therapy with four drugs is equally effective for eradication of H pylori infection. *Clinical gastroenterology and hepatology: the official clinical practice journal of the American Gastroenterological Association* 2010; **8**(1): 36-41.e1.
